# Supplementary material for: Strategic deployment of solar photovoltaics for achieving self-sufficiency in Europe throughout the energy transition
Source: Nat Commun. 2025 Jul 7;16:6259. doi: 10.1038/s41467-025-61492-9 (PMC12234877; doi:10.1038/s41467-025-61492-9)
Supplement: Supplementary file 1 — Supplementary Information [file 41467_2025_61492_MOESM1_ESM.pdf]

Supplementary Information for Strategic deployment of solar photovoltaics for achieving self-sufficiency in Europe throughout the energy transition

Parisa Rahdan[1,2,*], Elisabeth Zeyen[3], Marta Victoria[1,2,4]

[1] *Department of Mechanical and Production Engineering and iCLIMATE Interdisciplinary Centre for Climate Change, Aarhus University, 8000, Aarhus, Denmark*

[2] *Department of Wind and Energy Systems, Technical University of Denmark, Elektrovej 325, 2800, Lyngby, Denmark*

[3] *Department of Digital Transformation in Energy Systems, Technische Universität Berlin, Einsteinufer 25 (TA 8), 10587, Berlin, Germany*

[4] *Novo Nordisk Foundation CO_2 Research Center, Gustav Wieds Vej 10, 8000, Aarhus, Denmark*

[*] *Lead contact and corresponding author, Email: parra@dtu.dk*

Contents

Supplementary Note 1: PyPSA-Eur model and self-sufficiency constraint **2**

Supplementary Note 2: Carbon budget calculation **4**

Supplementary Note 3: Cost and land-use calculations for alternative solar configurations **5**

1. Inverter dimensioning 5
2. Delta configuration 7

Supplementary Note 4: Solar generation for different panel configurations **8**

Supplementary Note 5: Sensitivity analysis **10**

1. Transmission expansion allowance 10
2. Wind turbine modeling 11

Supplementary Figures **14**

Supplementary Tables **39**

Supplementary Note 1: PyPSA-Eur model and self-sufficiency constraint

We briefly go over some of the general features of the open-source PyPSA-Eur model here as detailed information regarding the optimization, the constraints, data sources, calculation of different demands, and assumptions made for modeling various technologies is already available in previous studies[1,2] as well as the model documentation[3]. The main objective function for the optimization is to minimise the total annualized system costs, as shown in Supplementary Equation (1).

$$\min_{G,F,E,P,g,f} = \Bigg[\sum_{i,r} c_{i,r}.G_{i,r} + \sum_{k} c_k.F_k + \sum_{i,s} c_{i,s}.E_{i,s} + \sum_{l} c_l.P_l + \sum_{i,r,t} w_t \left(\sum_{i,r} o_{i,r}.g_{i,r,t} + \sum_{k} o_k.f_{k,t}\right)\Bigg] \tag{1}$$

where c_* is capital cost of the component, o_* is operating cost of the component, $G_{i,r}$ is generator capacity of technology r at location i, $E_{i,s}$ is energy capacity of storage s at location i, P_l is transmission line capacity for line l, F_k is power capacity of technology k for conversion and transportation of energy, $g_{i,r,t}$ is generator dispatch of technology r at time t, and $f_{k,t}$ is dispatch of technology k at time t. Each time snapshot t is weighted by the time-step w_t, and the sum of time-steps is one year. Costs for all technologies and the source for each data are available at the GitHub repository of PyPSA Technology Data[4].

The assortment of linear constraints added to the optimisation problem is meant to represent different physical and societal limitations in the real-world energy system such as the maximum renewable potential in every region based on land availability, maximum transmission expansion limit based on social acceptance, available renewable and non-renewable resources depending on the weather, and maximum discharging and charging rates for each storage technology. These constraints vary from general to very specific, such as what percentage of the electric vehicles in the system can be used as batteries in each time-step. Other constraints in the model can help define a scenario's goals, such as the carbon emissions target or limiting the usage of gas in the system.

The self-sufficiency constraint, as shown in the main text, is meant to ensure that each country is able to produce a certain share of its own demand during the year. This constraint does not limit energy transmission, and when applied to the sector-coupled scenarios, does not differentiate between different energy carriers. To clarify how this constraint is implemented for various sectors, we can look at Supplementary Fig. 1. This figure shows a simplified representation of how the electricity sector is modeled for each node. The local production of electricity is the sum of generation by the various technologies while system losses happen due to the cyclic efficiency of batteries, pumped hydro storage units, and hydrogen conversion technologies. Already we can see

that including all these elements in a constraint is not a simple task. However, addition of other sectors and the interaction of energy carriers with each other complicates this even further.

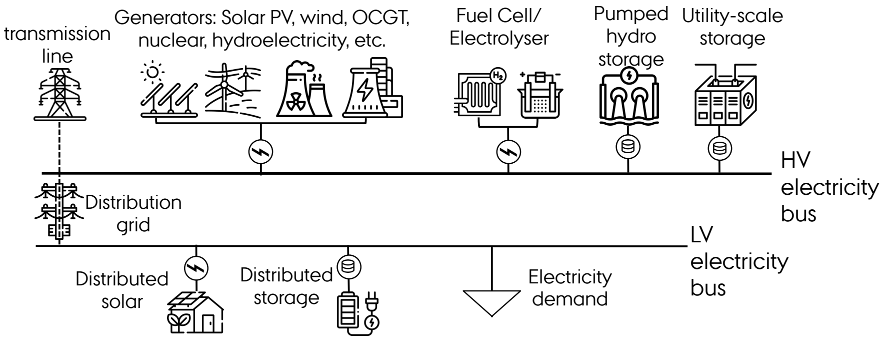

Supplementary Fig. 1: Modeling the energy system. Simplified representation of a single node when modeling the electricity sector. This figure includes icons designed by Iconjam (utility battery), NeXore88 (Fuel Cell), and freepik (all other icons) from flaticon.com.

Supplementary Fig. 2 shows how the gas bus and the hydrogen bus in each node are connected to electricity and heat generators, storage technologies, pipelines to other nodes, separate demands such as gas for industry, and finally to each other in the form of chemical processes. Calculating all the losses for such a system is immensely difficult and will have to include conversion efficiencies from not just the gas and hydrogen technologies, but also ones for other carriers including oil, solid biomass, and methanol. Therefore, the constraint is modified as shown in the main text to instead limit the net imports of each country. It stands to reason that if a country is obliged to produce energy equal to its own demand during the year, the net imports of it would be lesser than or equal to zero. Using this method when implementing the constraint for Supplementary Fig. 2, we again consider generators including open-cycle and closed-cycle gas turbines (OCGT and CCGT), and gas boilers for local production of energy, and calculate the net imports into the node using the hydrogen and gas pipelines. The production of fuel with chemical processes such as Fischer-Tropsch and methanolisation is also considered as local energy production in the constraint.

For the main model, the forms of energy transport also include electricity transmission, biomass transport, and oil or methanol exchange. Similar to pipelines and transmission network, biomass transport includes a cost in our model, assuming to be done by trucks, but there is no upper limit to the capacity of biomass transport. Both methanol and oil exchange between countries are assumed to be done with negligible costs and without any limitations. It should be noted that this is still a computationally heavy constraint, and solving an optimisation problem with high spatio-temporal resolution under this constraint can take up to several hours and 100 GB of memory to complete.

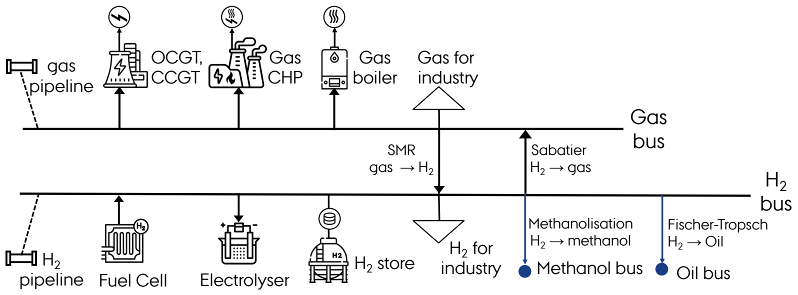

Supplementary Fig. 2: Modeling different energy carriers. Simplified representation of the gas and hydrogen bus of a single node when modeling all the sectors. For detailed information on different processes within the system refer to Neumann et al.[1]. This figure includes icons designed by NeXore88 (Fuel Cell) and freepik (all other icons) from flaticon.com.

Supplementary Note 2: Carbon budget calculation

The carbon budget for Europe is calculated by taking the global carbon budget estimated to be left by 2025 for a temperature increase of 1.7°C, distributing it among countries by assuming equal per capita emissions, and including EU27 countries plus Norway, Switzerland, and the UK. The carbon budget left for Europe by 2020 is 45 Gt[5], and based on carbon emissions data for 2020-2022[6,7], plus estimated carbon emissions for 2023 and 2024, the budget left for 2025 onwards is equal to 29 Gt. This budget is allocated to different time steps following an exponential decay with carbon neutrality imposed in 2050 (Supplementary Fig. 3)[8]. Note that the carbon budget assumed only includes emissions from sectors that are included in the model, and the total emissions for 2023 and 2024 are predicted with a conservative assumption regarding the yearly decrease in emissions.

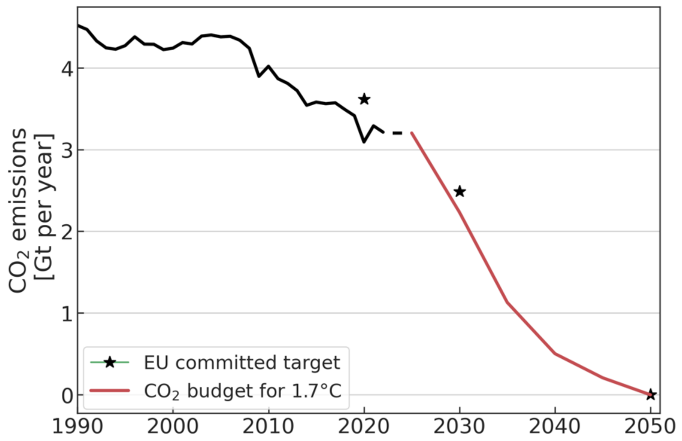

Supplementary Fig. 3: Annual carbon emissions. The carbon budget assumed for Europe is equal to 29 Gt (+1.7°C). Carbon neutrality is required by 2050.[1]

Supplementary Note 3: Cost and land-use calculations for alternative solar configurations

1. Inverter dimensioning

The capital cost, expressed in $(\frac{€}{\mathrm{MW_{AC}}})$, of a PV installation with an inverter DC/AC ratio equal to r is calculated using Supplementary Equation (2). In a PV installation, one part of the capital cost depends on the DC capacity (e.g. PV modules, land-use costs, soft costs), represented in Supplementary Equation (2) as DC components capital cost$_{\frac{\mathrm{DC}}{\mathrm{AC}}=1}$, while the other part depends on the AC capacity of the plant (e.g. inverter, grid-connection), represented as AC components capital cost$_{\frac{\mathrm{DC}}{\mathrm{AC}}=1}$. Therefore, for a PV power plant with a DC/AC ratio of r :

$$\text{PV power plant capital cost}_{\frac{\mathrm{DC}}{\mathrm{AC}}=r}(\frac{€}{\mathrm{MW_{AC}}}) =$$

$$\text{DC components capital cost}_{\frac{\mathrm{DC}}{\mathrm{AC}}=1}(\frac{€}{\mathrm{MW_{DC}}}) \cdot r\ (\frac{\mathrm{MW_{DC}}}{\mathrm{MW_{AC}}}) + \text{AC components capital cost}_{\frac{\mathrm{DC}}{\mathrm{AC}}=1}(\frac{€}{\mathrm{MW_{AC}}}) \tag{2}$$

To see the impact of the new calculation method, which incorporates inverter sizing, consider the following example: In previous studies using PyPSA-Eur, the investment cost for utility-scale PV in 2030 was assumed to be €380.4/kW_AC, based on DEA[9]. However, this cost corresponds to a module with a DC/AC ratio of 1.25. This detail can be easily overlooked, as pairing a 1 kW panel with a 0.8 kW inverter results in less than a 3% loss in annual solar generation (see Fig. 4). By applying Supplementary Equation (2), the investment cost for utility-scale solar PV is recalculated to €320.8/kW_AC (€251.3 for the DC parts + €69.5 for the AC parts), effectively reducing the investment cost by 15.8%.

Detailed assumptions for DC and AC components' capital cost for different investment years is shown in Supplementary Table 1. The final capital cost for fixed panels and horizontal single-axis tracking (HSAT) with different inverter ratios as calculated by Supplementary Equation (2) is also shown in Supplementary Table 1. According to the Technology Data Catalogue[9] published by the Danish Energy Agency (DEA), the share of AC components cost amounts to 19% of total PV power plant capital cost in 2020. This percentage might increase in the future as PV modules are reducing their cost at a faster rate than inverters and other grid-related costs. The DEA estimates the share to be 23% by 2050.

Supplementary Table 1: Capital cost assumption for fixed panels and HSAT with different inverter ratios for 2025-2050.

Technology	DC components cost (€/kW$_{DC}$) for 2025-2030-2040-2050	AC components cost (€/kW$_{AC}$) for 2025-2030-2040-2050	DC/AC ratio	Capital cost (€/kW$_{AC}$) for 2025-2030-2040-2050	Land use ($MW_{DC}\ km^{-2}$)
Fixed panels	313.7 - 251.4 - 208.2 - 189.0	80.9 - 69.5 - 60.5 - 56.3	1	394.7 - 320.9 - 268.8 - 245.3	102
			1.3	488.6 - 396.3 - 331.2 - 302.1	78.5
			1.5	551.2 - 446.6 - 372.9 -339.8	68
			1.7	613.8 - 496.8 - 414.5 - 377.6	60
			1.9	676.4 - 547.1 - 456.2 - 415.5	53.7
HSAT	377.2 - 307.9 - 258.9 - 236.9	80.9 - 69.5 - 60.5 - 56.3	1	458.1 - 377.5 - 319.6 - 293.3	88.8
			1.3	571.1 - 469.9 - 397.3 - 364.4	68.3
			1.5	646.4 - 531.4 - 449.1 - 411.8	59.3
			1.7	721.8 - 593.0 - 500.8 - 459.2	52.2
			1.9	797.1 - 654.6 - 552.7 - 506.5	46.7

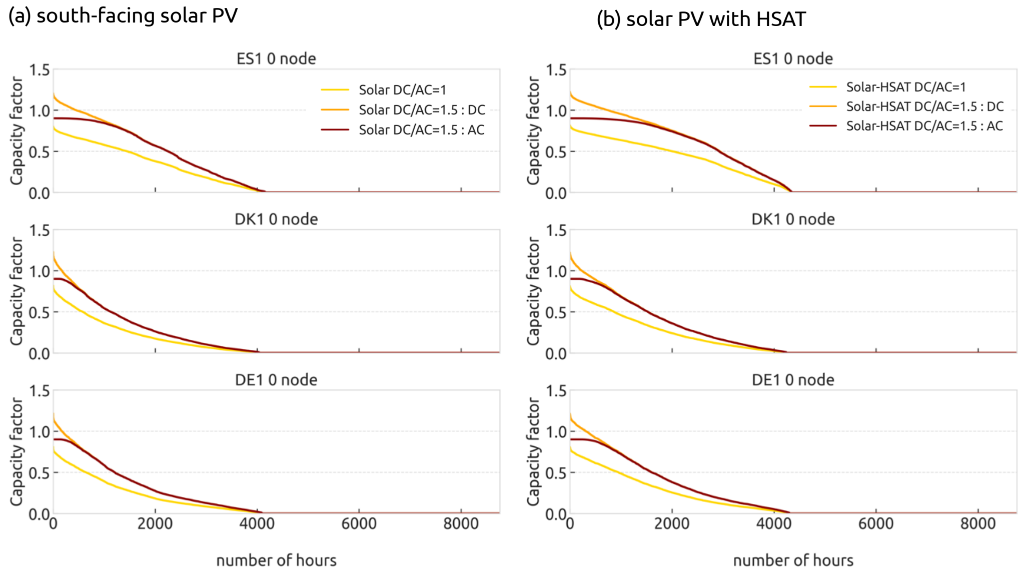

Supplementary Fig. 4: Effect of inverter dimensioning. Duration curve of capacity factors for (a) south-facing fixed solar PV with DC/AC ratio of 1 and 1.5 (both DC and AC generation are shown), and (b) solar PV with horizontal single-axis tracking (HSAT) with DC/AC ratio of 1 and 1.5 (both DC and AC generation are shown).

2. Delta configuration

There is very limited information regarding the costs and land use of delta-shaped or east-west PV plants in literature[10]. Therefore, we conduct our own calculations for these configurations. For land use, considering the higher packing factor for direct land use, and assuming direct land use accounts for 77% of total land use for the plant[11], 30% higher capacity density, relative to south-oriented systems, is assumed for the delta configuration.

As for costs, we follow a similar approach as the calculation done for inverter dimensioning. We assume each 1 kW of PV panels in the delta configuration is paired with a 0.66 kW inverter (this is the same as having a 1.5 DC/AC ratio). We estimate that the lower cost of the inverter and the lower land-use will result in a system that is 27% cheaper than south-oriented systems, resulting in a capital cost equal to €250/kW_AC (€320.8/kW_AC ∗ 0.78).

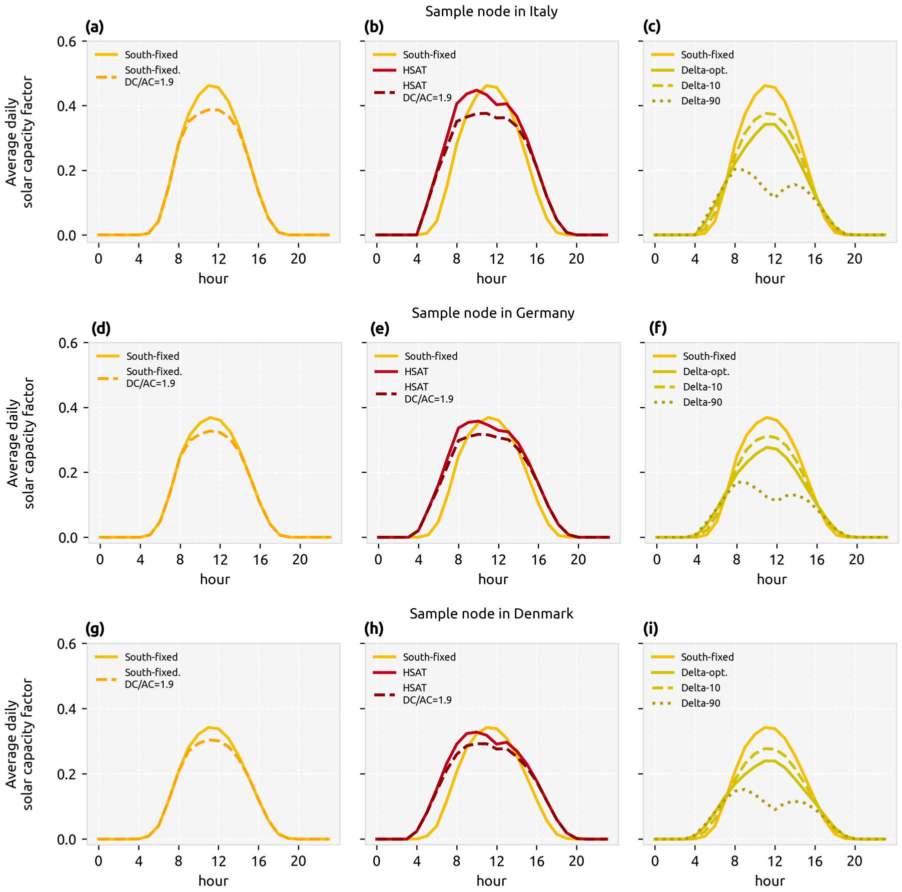

Supplementary Fig. 5: Comparison of solar PV configurations. Average daily capacity factors for sample nodes in Germany, Italy, and Denmark throughout the year for (a,d,g) solar PV with fixed panels facing south and country-wise optimal slope with 1 and 1.9 inverter DC/AC ratio, (b,e,h) solar PV with horizontal single-axis tracking with 1 and 1.9 inverter DC/AC ratio, and (c,f,i) delta configuration with 10°/optimal/90° inclination. The optimal inclination here refers to the best inclination for the south-facing panel that is optimised for each country.

Supplementary Note 4: Solar generation for different panel configurations

We briefly look at the maximum available solar energy for panels with different inclinations and orientations. As seen from Supplementary Fig. 6, the optimal orientation for a solar panel is always south, with an inclination between 30° and 35°. But looking at how much of the load can be covered by solar generation at every hour, east-facing panels are superior to west-facing ones. This is also true if we consider how much of the load can be covered by solar generation for the six hours with the highest electricity demand in each day. The higher load coverage for east-facing panels is due to higher solar generation in morning, as shown in Supplementary Fig. 7. The higher average solar radiation in the morning in Europe is the main cause behind the superiority of east-facing panels (also seen in Fig. 2 of main text), which could be due to better average weather conditions. The non-symmetry of solar radiation is observed in other weather years as well.

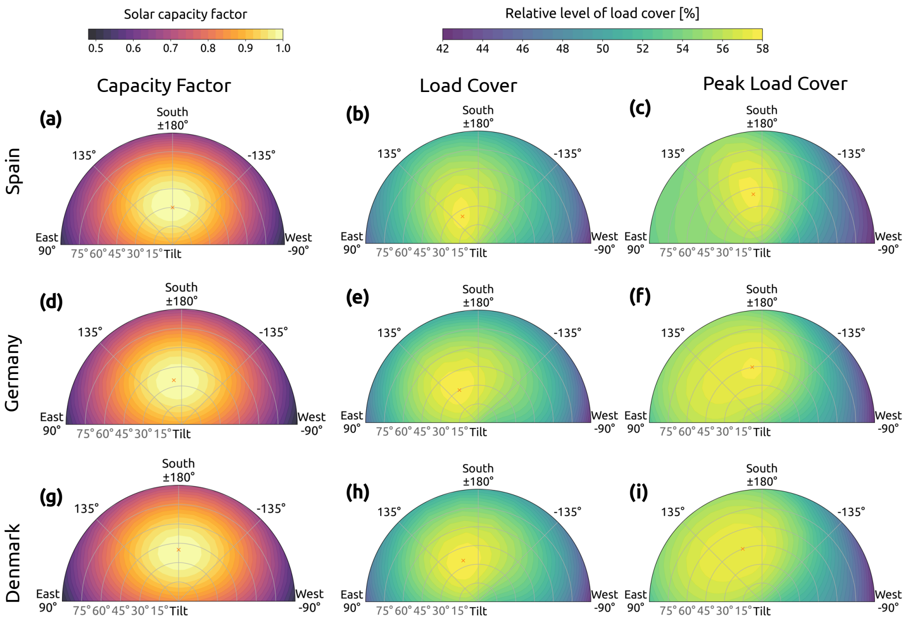

Supplementary Fig. 6: Optimal solar PV tilt and orientation. (a,d,g) Annual capacity factor for different combinations of tilt and orientation angles for selected countries. Both radiation and the effect of temperature on panel efficiency are accounted for when calculating the capacity factor. The ratio of (b,e,h) total demand and (c,f,i) sum of demand for peak 6-hour period that can be covered by solar generation with a fixed capacity under different combinations of tilt and orientation angles for selected countries. The fixed capacity is calculated by assuming the average demand is equal to the solar generation of a south-facing panel with 35° tilt. Figures were generated using ERA5 reanalysis data for year 2013.

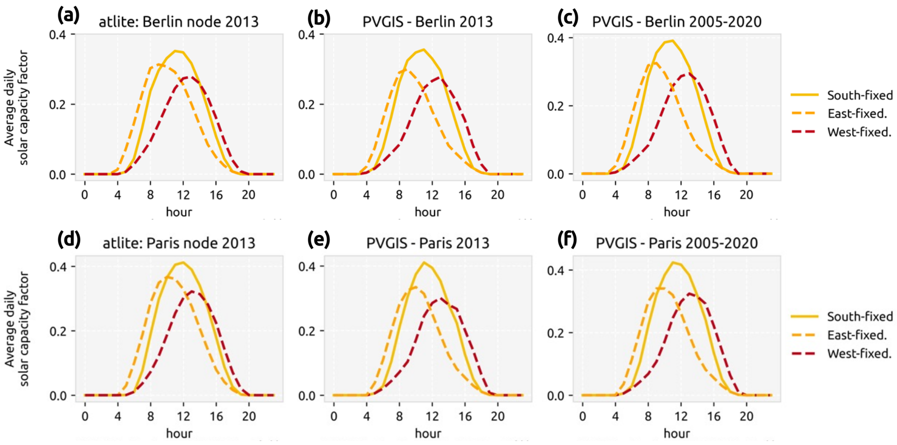

Supplementary Fig. 7: East vs. South vs. West facing solar PV. Hourly capacity factor for a day (average throughout the year or years) for the Berlin and Paris nodes: (a,d) as calculated by the atlite package using radiation data from satellite-aided SARAH-2 dataset and temperature data from ERA5 reanalysis dataset for weather year 2013, compared with values for the same locations in (b,e) 2013 and (c,f) 2005 to 2020 from the SARAH-2 dataset as calculated by PVGIS web interface. All figures indicate a higher PV production in the morning than in the evening, but the bias from atlite is higher than the PVGIS data for 2013[12–14]. The higher production in morning hours was also seen in the results of Szabo at al. when investigating vertical bifacial PV systems in Europe[15]. Note: the timestamp variation between ERA5 data and SARAH-2 dataset has been accounted for in the current study by shifting the SARAH-2 data by -30 minutes. For more details refer to PVGIS documentation on what the timestamps in each dataset represent[16].

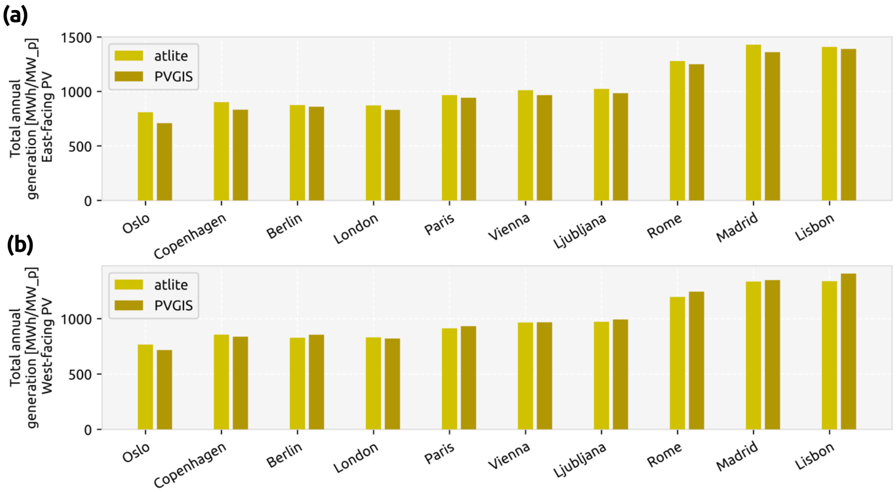

Supplementary Fig. 8: East vs. West facing solar PV. Comparison between total annual generation of (a) East-facing and (b) West-facing PV panels with 10° tilt calculated by atlite and using data from PVGIS for 10 locations in Europe, averaged over 18 years from 2005 to 2023. Furthermore, results from Alkan et. al[17] confirm that generation from PVGIS matches well with experimental results from a 170 kW_p rooftop delta PV installation located in Istanbul, Turkey.

Supplementary Note 5: Sensitivity analysis

1. Transmission expansion allowance

The transmission network expansion is limited to 10% of today's capacity (calculated based on existing and planned lines by ENTSO-E[18]) in all scenarios presented in the main text. This conservative assumption reflects the currently slow pace of transmission line development and the challenges of public acceptance for grid expansion across Europe[19]. To assess the impact of higher transmission expansion rates, we conduct a sensitivity analysis. Previous studies have demonstrated that greater flexibility in the transmission network leads to higher wind deployment[1,20]. This is because, unlike solar, which is relatively homogeneous across regions, wind generation exhibits strong spatial variability, even between neighboring areas, making electricity transmission more valuable for wind energy. As shown in Supplementary Fig. 9, higher transmission expansion reduces the solar-to-wind generation ratio by 2–10%. This does not significantly alter the system's generation mix, and innovative solar configurations, such as horizontal single-axis tracking (HSAT) and inverter dimensioning, remain cost-efficient even under unrestricted transmission expansion. Additionally, other findings, such as the higher system costs under self-sufficiency constraints for typically net-importing countries, also remain valid.

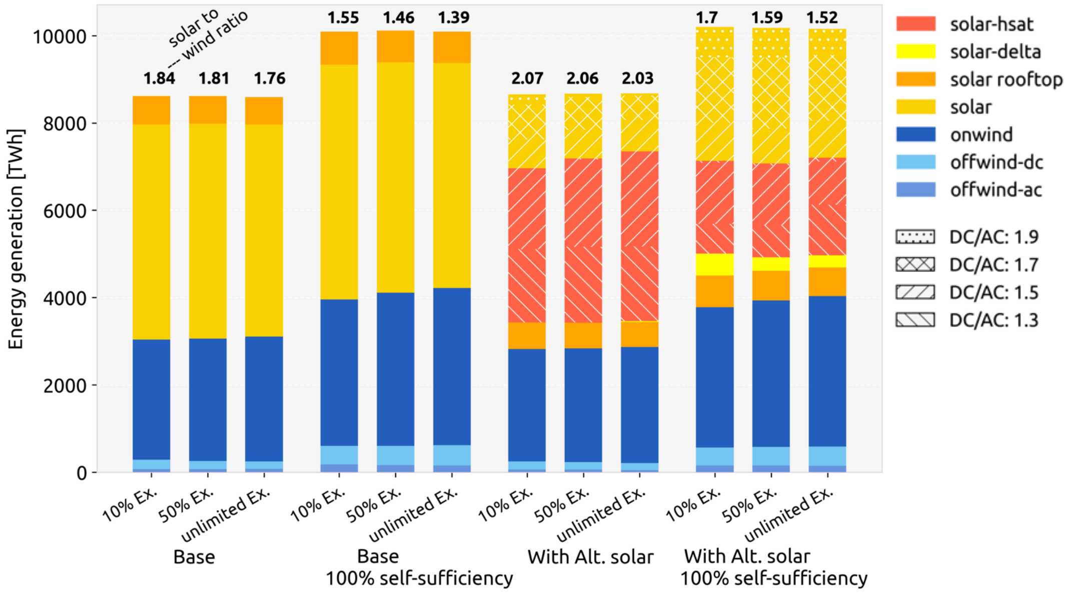

Supplementary Fig. 9: Comparison of energy generation of major renewable technologies for overnight runs. The runs represent the four main scenarios (Base, Base with 100% self-sufficiency target, Alternative solar configurations, and Alternative solar configurations with 100% self-sufficiency target) under different transmission expansion allowances.

2. Wind turbine modeling

In the second sensitivity analysis, we explore whether improving wind turbine modeling could have a similar impact to introducing new solar configurations. In our model, each wind turbine is characterized by a power curve (Supplementary Fig. 10), which determines potential energy output at each time-step for 370 regions, based on wind velocity data from the ERA5 reanalysis dataset for the 2013 weather year. The VESTAS-3MW is the default wind turbine used for modeling Onshore wind turbines in our model.

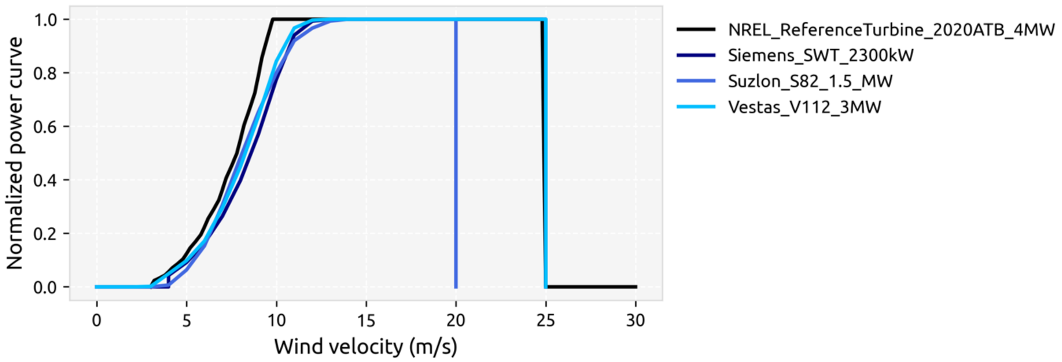

Supplementary Fig. 10: Comparison of wind turbines. Normalized power curves for 4 different onshore wind turbines as provided by the atlite package[21]. Each power curve can be used to add a new type of turbine to the model

To enhance the model, we introduce the 'NREL-4MW' turbine, sourced from the NREL ATB 2020 database[22], as an additional wind technology in overnight scenarios under two different transmission expansion assumptions. Based on projected turbine cost data for 2030 provided by NREL Annual Technology Baseline (ATB 2020)[22], and assuming the same costs for other expenses of the wind plant as those provided by DEA[9] (which are used for the default turbine), the NREL-4MW would be 16%-58% more expensive than the default wind turbine. Since detailed cost data for this turbine is unavailable, we conduct the sensitivity analysis using two cost scenarios: one where the NREL-4MW and VESTAS-3MW turbines have the same investment costs as an optimistic approach, and another where the NREL-4MW has 44% higher costs than the VESTAS-3MW. Both scenarios are modeled with unlimited transmission expansion.

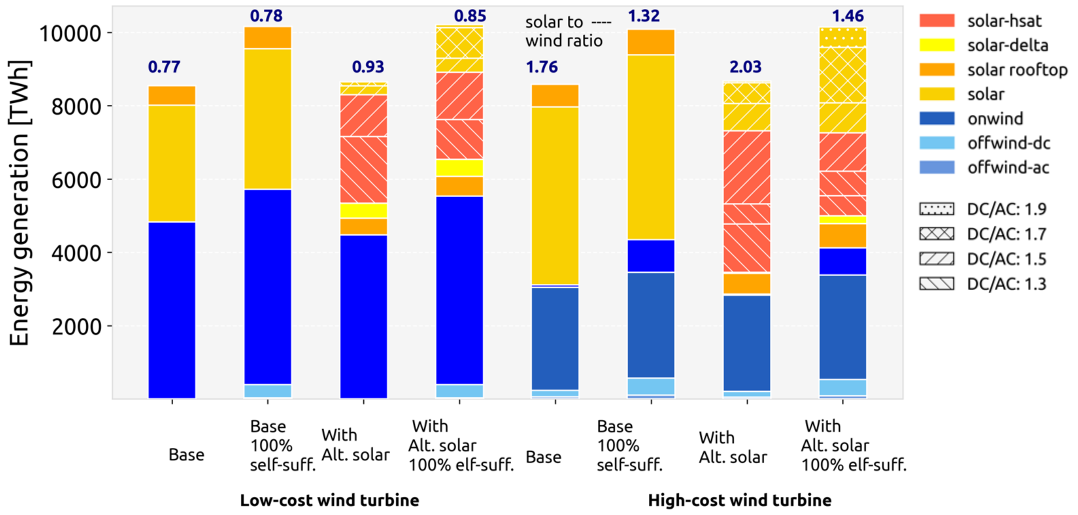

Supplementary Fig. 11: Comparison of energy generation of major renewable technologies for overnight runs. The runs represent the four main scenarios (Base, Base with 100% self-sufficiency target, Alternative solar configurations, and Alternative solar configurations with 100% self-sufficiency target) with unlimited transmission expansion allowance and including a new wind turbine (NREL 4-MW) with enhanced power curve under two different cost assumptions

In the "low-cost" scenario, the NREL-4MW onshore wind turbine—capable of generating up to 30% more power than the VESTAS-3MW in some regions—becomes the dominant wind technology across all areas, shifting the system toward increased onshore wind capacity. As a result, the solar-to-wind ratio decreases by 51% in the Base scenario. However, when the NREL-4MW turbine's costs are 44% higher, the outcomes closely resemble those of the main scenarios, with a maximum reduction of 6% in the solar-to-wind ratio compared to the scenarios shown in Fig. 9 with unlimited expansion.

Therefore, the energy mix could undergo substantial changes if a higher-yielding or more cost-effective onshore wind turbine becomes available. Future studies could build on this analysis by incorporating more precise cost estimates for a broader range of commercially available wind turbines.

Supplementary Table 2: Comparison between the ratio of total installed solar capacity (utility and rooftop) to total installed wind capacity (onshore wind and offshore) in the Base overnight scenario of this study to recent similar studies.

Study	Main assumptions	Solar to wind ratio (Installed capacity)
**Base overnight scenario**	Sector-coupled, 95% emissions reduction, 2050 cost assumptions, 10% grid expansion	1.84
**Gotske at. al (2024)**[20]	Sector-coupled, net-zero target, 2030 cost assumptions, average of 60 weather years, no grid expansion (see Supp. Figure 39)	1.9
**Neumann at. al (2023)**[1]	Sector-coupled, net-zero target, 2030/2050 cost assumptions*, zero/unlimited grid expansion	2030 costs: 1.6 (0)/1.4 (unlim.)<br>2050 costs: 2.7(0)/2.2 (unlim.)
**Gawlick & Hamacher (2023)**[19]	Electricity and hydrogen sectors only, net-zero target, 2050 cost assumptions from IEA**, unlimited grid expansion (see Table A.9, 'totally flexible' scenario)	1.6

* 2050 assumptions include cost reductions of utility solar by 25% and onshore wind by 7% relative to 2030

** Investment cost ratio for onshore wind to solar is 3.5 based on this data, 3.4 based on DEA without consideration of inverter dimensioning, and 4.1 when reducing solar cost by considering inverter dimensioning

Supplementary Table 3: Comparison between the ratio of solar (utility and rooftop) to wind (onshore wind and offshore) in terms of installed capacity and energy generation for the Base myopic scenario of this study to recent similar studies.

Study	Main assumptions	Solar to wind ratio (Energy generation)	Solar to wind ratio (Installed capacity)
**Base Myopic scenario**	2050 with net-zero target under 1.7° temperature increase carbon budget starting from 2025	1.69	3.98
**Zeyen at. al (2023)**[5]	2050 with net-zero target under 1.7° temp. increase budget from 2020 (Fig. S31)	0.65 (ranging from 0.39 to 0.77 with different learning rates for solar, wind, and electrolysis)	1.36 (ranging from 0.85 to 1.69)
**Bogdanov et. al (2019)**[23]	2050 with net-zero target, global scenario (Supp. Table 12)	1.48	3.5
**Breyer at. al (2023)**[24]	2050 with net-zero target, sector-coupled for Europe, scenarios: moderate /leading (Table 1)	1.88 / 1.92	4.7 / 4.5

Supplementary Figures

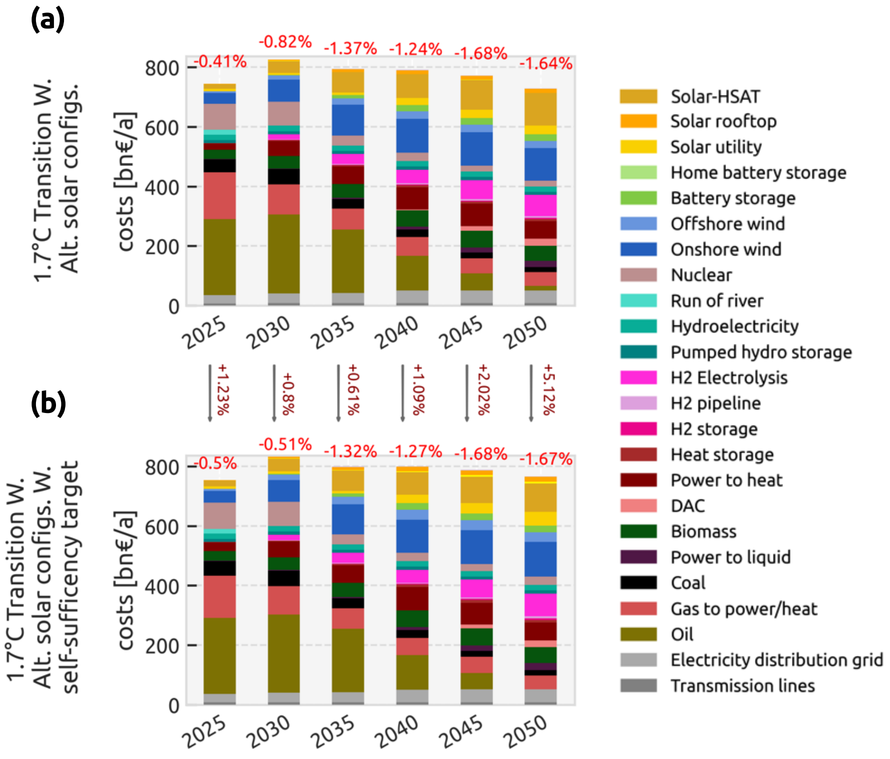

Supplementary Fig. 12: Changes in total system costs during the transition under a 1.7°C temperature increase target with alternative solar configurations. Costs are shown for scenarios (a) without and (b) with self-sufficiency target. The red numbers on each figure show the decrease in total system costs for scenarios with alternative solar configurations relative to scenarios without them, which are shown in Fig. 1 of the main text. For example, in year 2025, adding alternative solar configurations reduces total system cost for the 1.7°C Transition with Self-sufficiency Target scenario by 0.5%. The total system cost is lowered by an average of 1.43% when alternative solar configurations are added to the base transition, and by an average of 1.39% when alternative solar configurations are added to the transition with self-sufficiency target. The discount rate used to calculate total cost throughout the whole transition is 7%. Detailed information on the capital cost and lifetime assumed for each technology are available in the GitHub repository of PyPSA Technology Data[4].

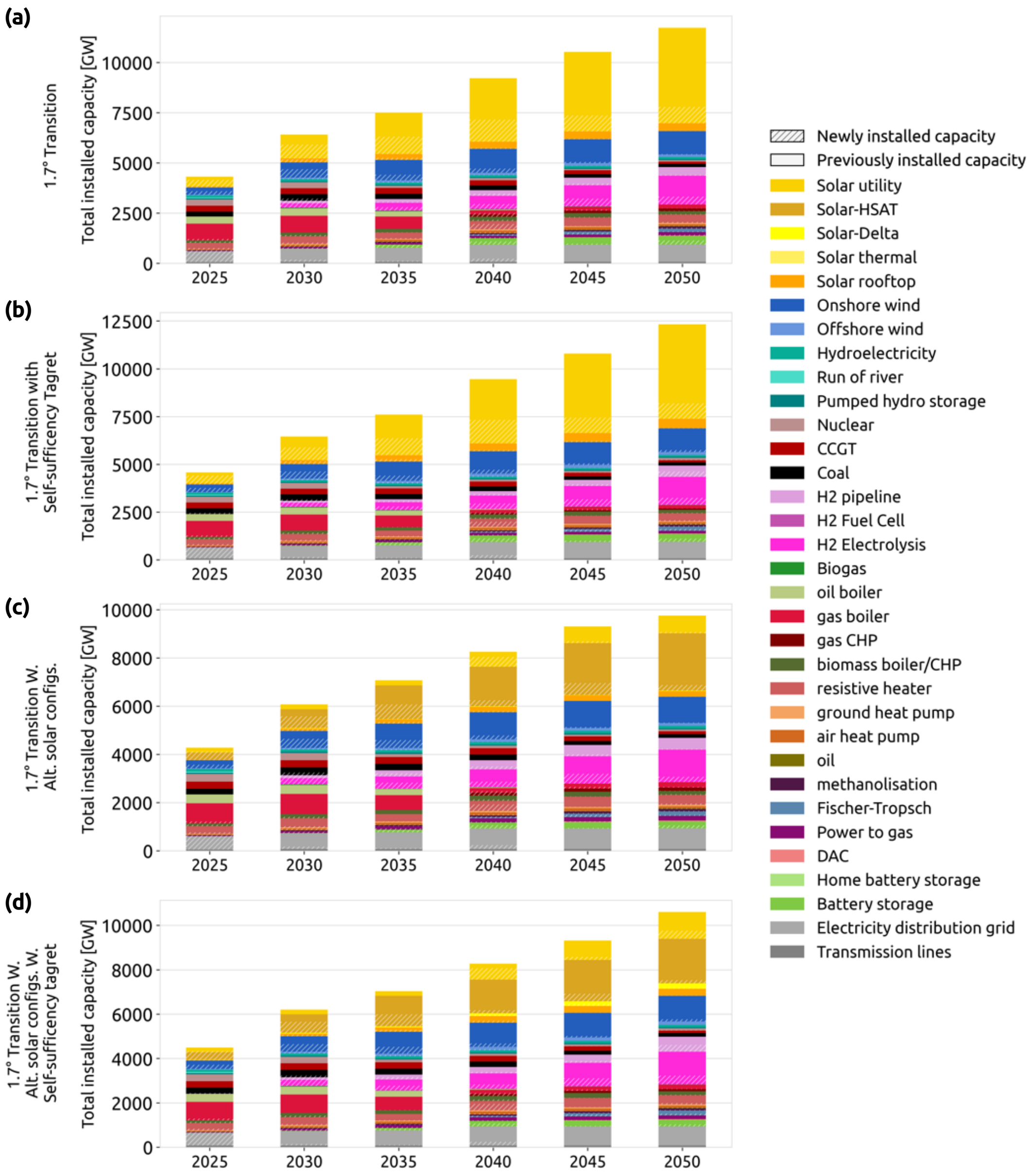

Supplementary Fig. 13: Changes in total installed capacity during the transition under a 1.7°C temperature increase target for all scenarios. The changes are shown for (a) Base scneario, (b) Base scenario with 100% self-sufficiency target, (c) Alternative solar configurations scneario, and (d) Alternative solar configurations scneario with 100% self-sufficiency target. The installed capacity in each investment period is divided into previously installed capacity (brownfield) and newly installed capacity. The previously installed capacity at the beginning of the transition (2025) is taken from datasets provided by IRENA [25].

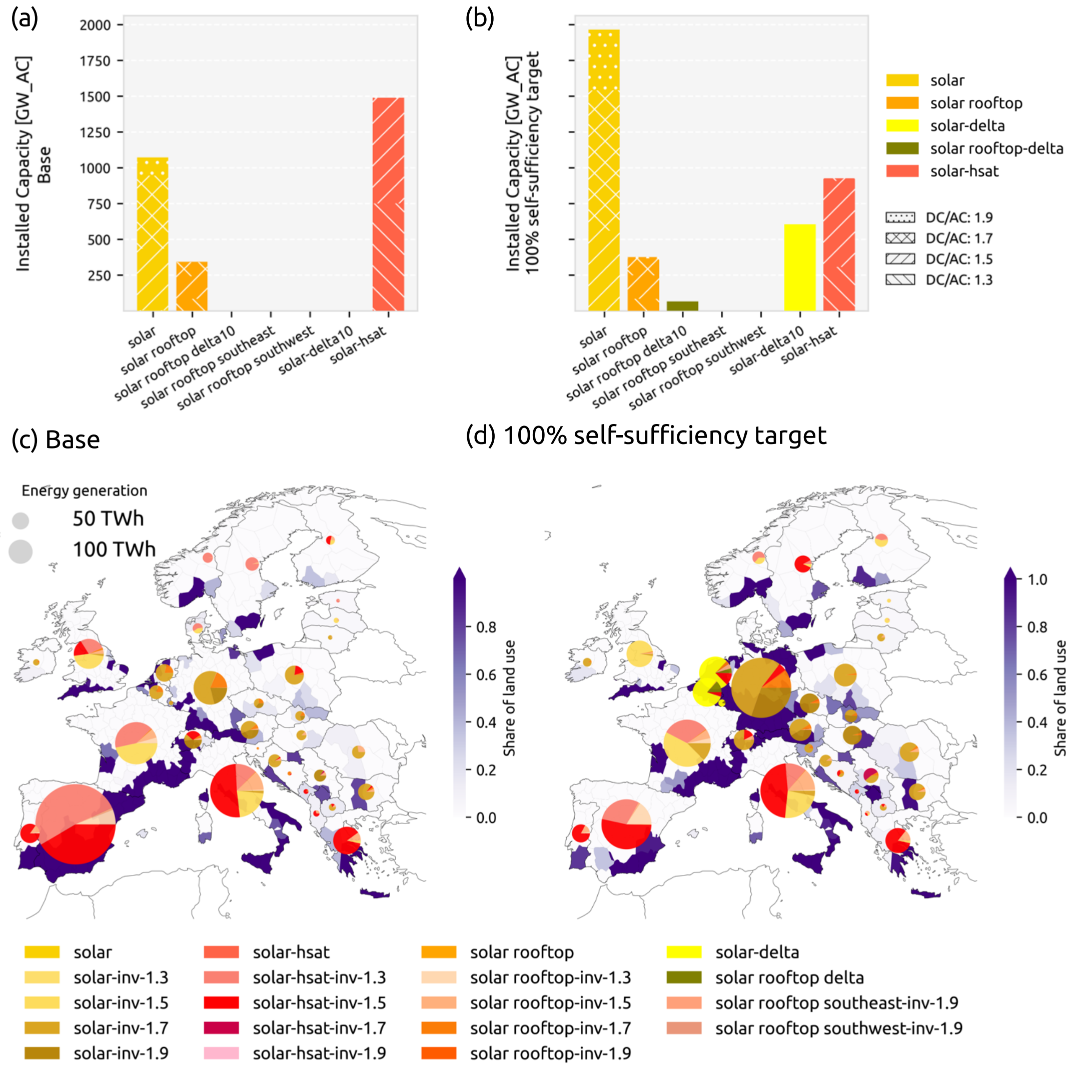

Supplementary Fig. 14: Comparison of the cost-efficiency of alternative solar technologies. Results for the overnight scenarios (95% carbon emissions reduction, greenfield assumptions, technology costs for 2050) where the cost-efficiency of 19 alternative solar configurations is examined. The installed capacity of all alternative configurations for the overnight scenarios is shown at (a) without self-sufficiency target and (b) with self-sufficiency target. The solar generation map for all alternative configurations for the overnight scenarios is shown at (c) without self-sufficiency target and (d) with self-sufficiency target.

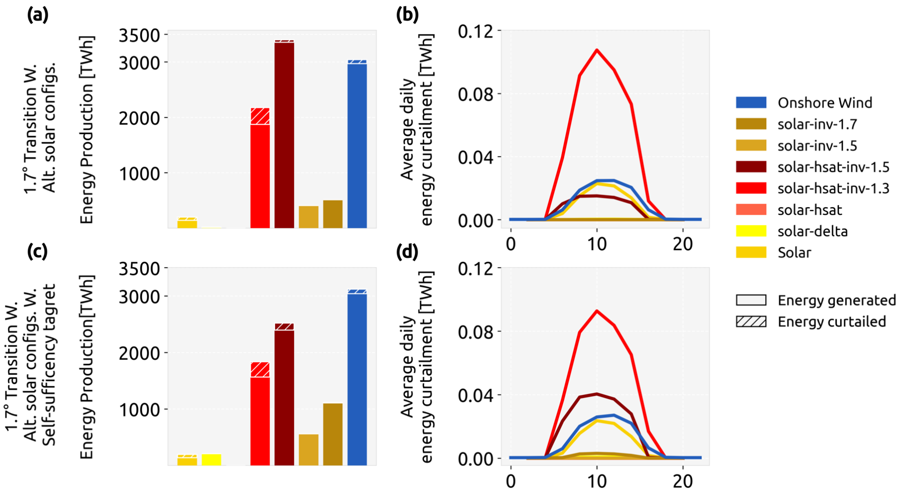

Supplementary Fig. 15: Comparison of total energy generation and curtailment for wind and solar. Results for the transition scenarios showing (a,c) Total energy generation from solar PV configurations and onshore wind plus the amount of curtailed energy, and (b,d) average daily energy curtailment. The high curtailment ratio of HSAT with DC/AC ratio of 1.3 indicates the extra energy at noon is not deemed necessary by the system. This is the reason HSAT with DC/AC ratio of 1.5 has a higher installed capacity than HSAT with DC/AC ratio of 1.3 in many countries, even though DC/AC ratio of 1.3 is more cost-efficient overall (refer to Fig. 2 of main text). As discussed in the main text, total generation vs. capital cost is not the only deciding factor when it comes to the selection of a configuration, as evidenced by the results shown in Fig. 3 and Fig. 4 of the main text.

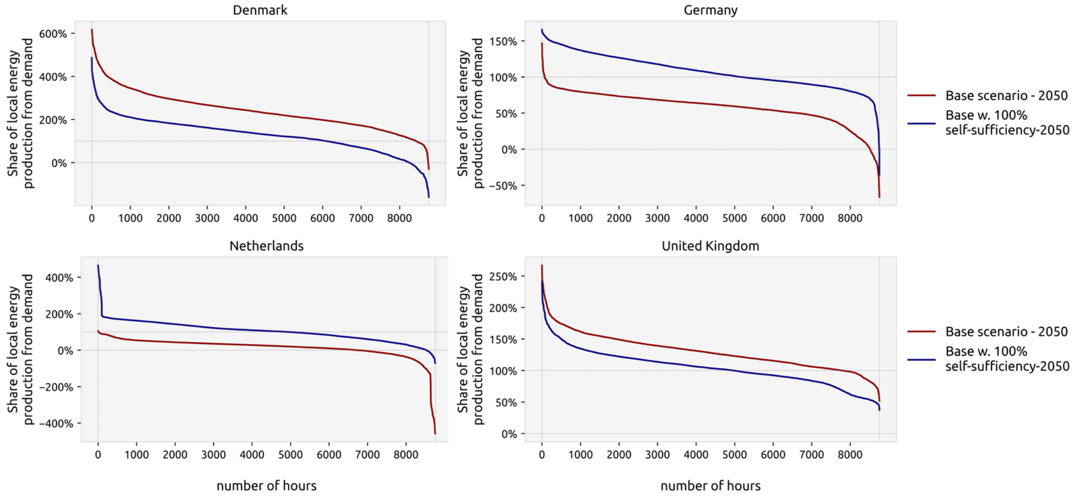

Supplementary Fig. 16: Duration curves for local energy production share for different countries. Each curve shows the share of local energy production from demand for all energy carriers for base scenario and base scenario with 100% self-sufficiency target in 2050.

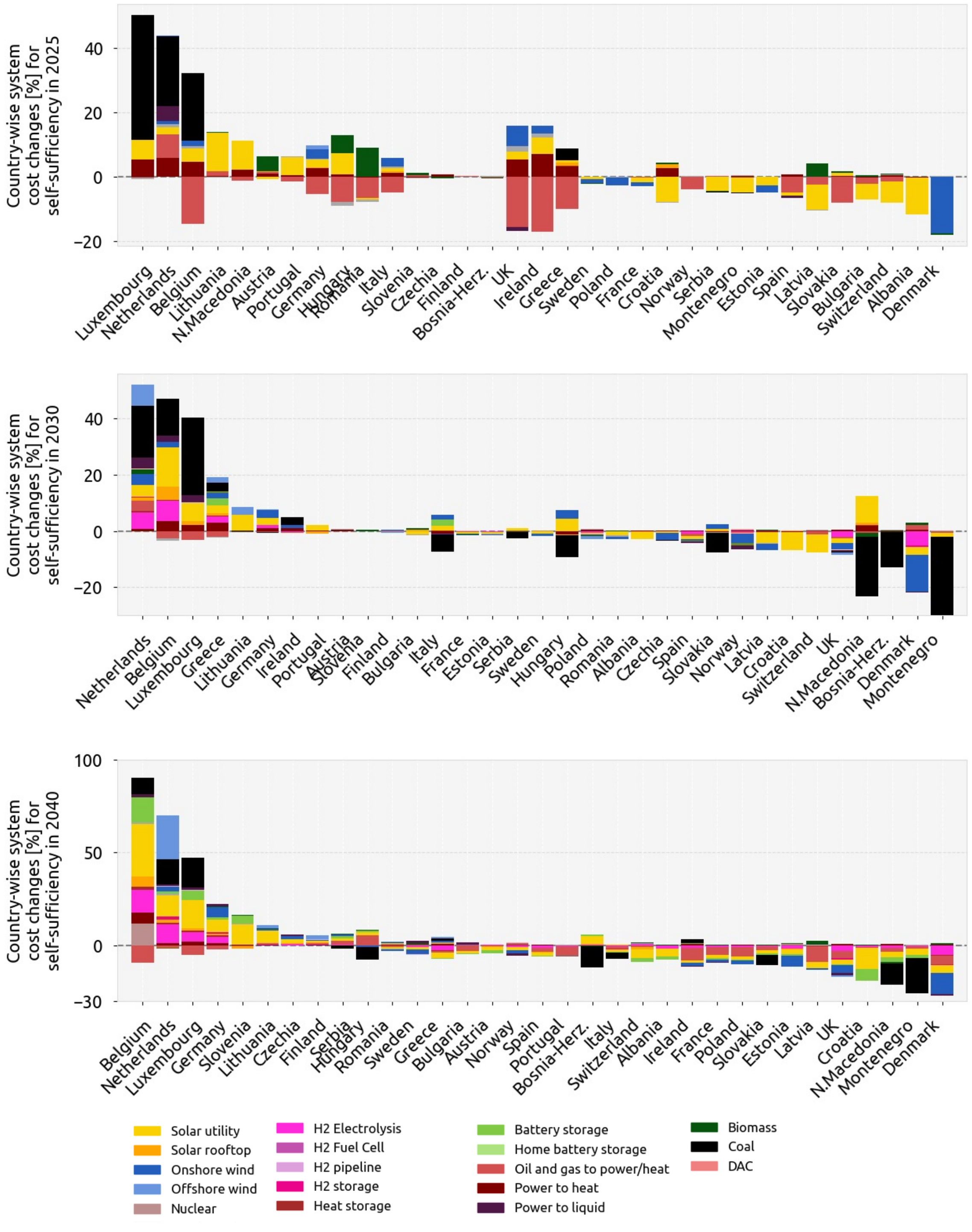

Supplementary Fig. 17: Country-wise total system cost changes under the self-sufficiency target for base transition scenarios. Changes are shown for the scenario with self-sufficiency target relative to one without it. The increase/decrease in total country costs is shown as a percentage, and the share of each component from this increase is also shown for each country. Countries in each figure are ordered from one with the highest increase to the one with the highest decrease in costs. Transmission lines, H2 pipelines, gas pipelines, and other components where the costs would be shared between countries are not shown in the figure.

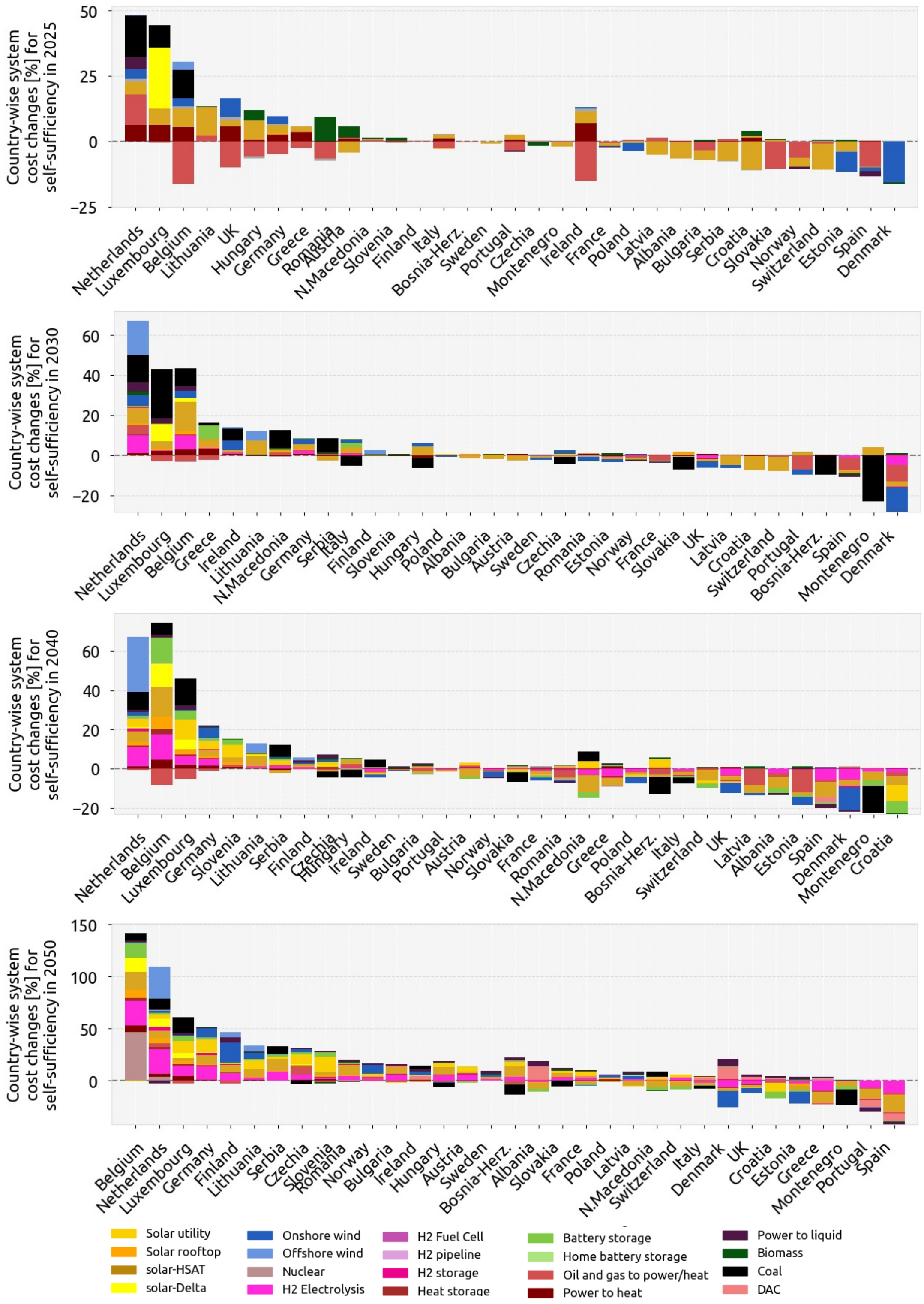

Supplementary Fig. 18: Country-wise total system cost changes under the self-sufficiency target for transition scenarios with alternative solar configurations. All scenarios include alternative solar configurations. Changes are shown for the scenario with self-sufficiency target relative to one without it. The increase/decrease in total country costs is shown as a percentage, and the share of each component from this increase is also shown for each country.

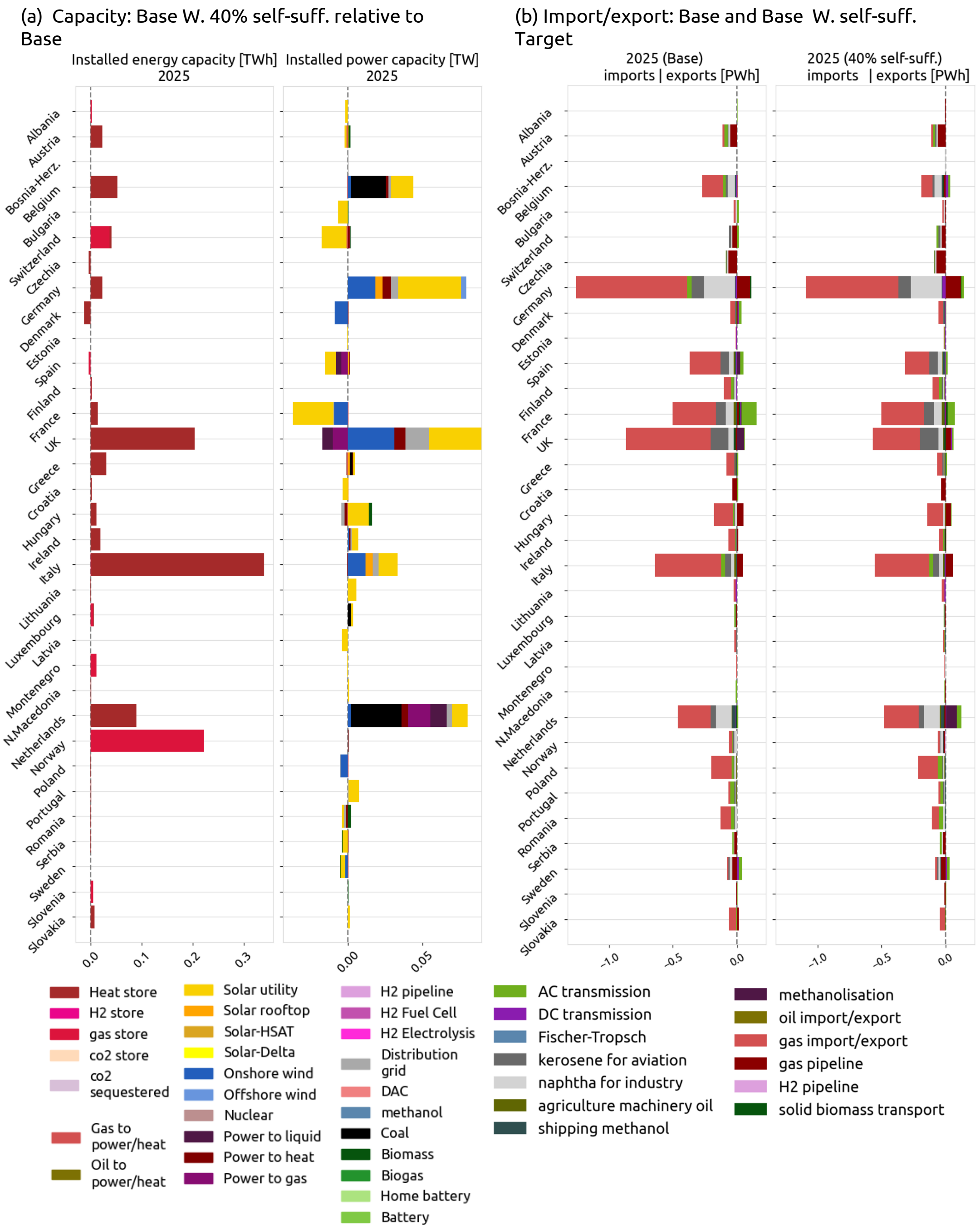

Supplementary Fig. 19: Effect of 40% self-sufficiency for Base scenarios. (a) Changes in the total installed capacity of different technologies for the base scenario with 40% self-sufficiency target relative to the base scenario in 2025. (b) Changes in import and export of different countries for the base scenario and base scenario with 40% self-sufficiency target in 2025. Installed energy capacity is shown for storage technologies and power capacity for the rest. Power to gas includes capacities that produce hydrogen using steam methane reforming (SMR) and methane using the Sabatier process[1]. The increase in the capacity of distribution grid under the self-sufficiency scenario is due to the higher electricity demand for heat pumps at the low-voltage bus due to the lower gas imports limiting the use of gas boilers to provide heating.

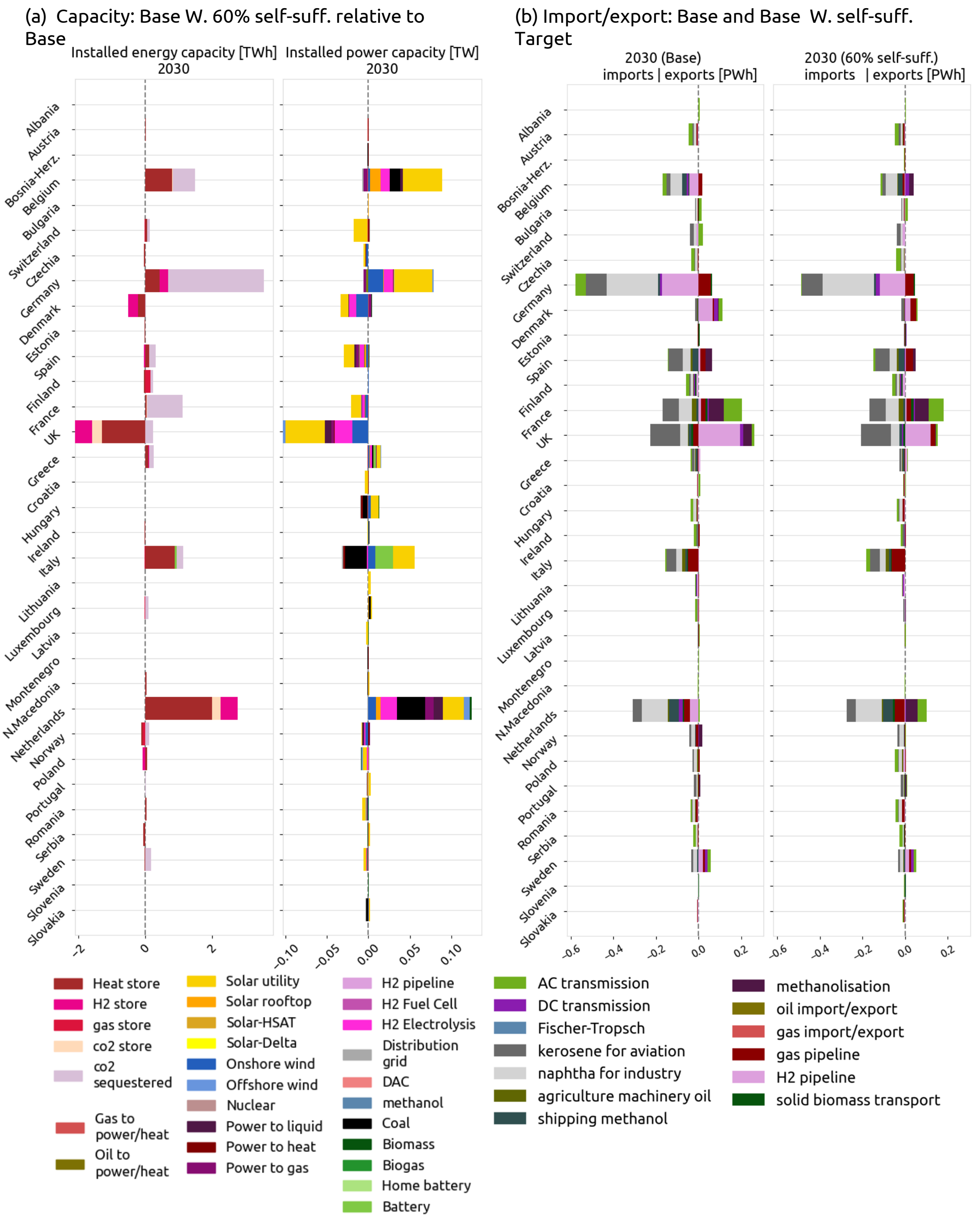

Supplementary Fig. 20: Effect of 60% self-sufficiency for Base scenarios. (a) Changes in the total installed capacity of different technologies for the base scenario with 60% self-sufficiency target relative to the base scenario in 2030. (b) Changes in import and export of different countries for the base scenario and base scenario with 60% self-sufficiency target in 2030. Installed energy capacity is shown for storage technologies and power capacity for the rest.

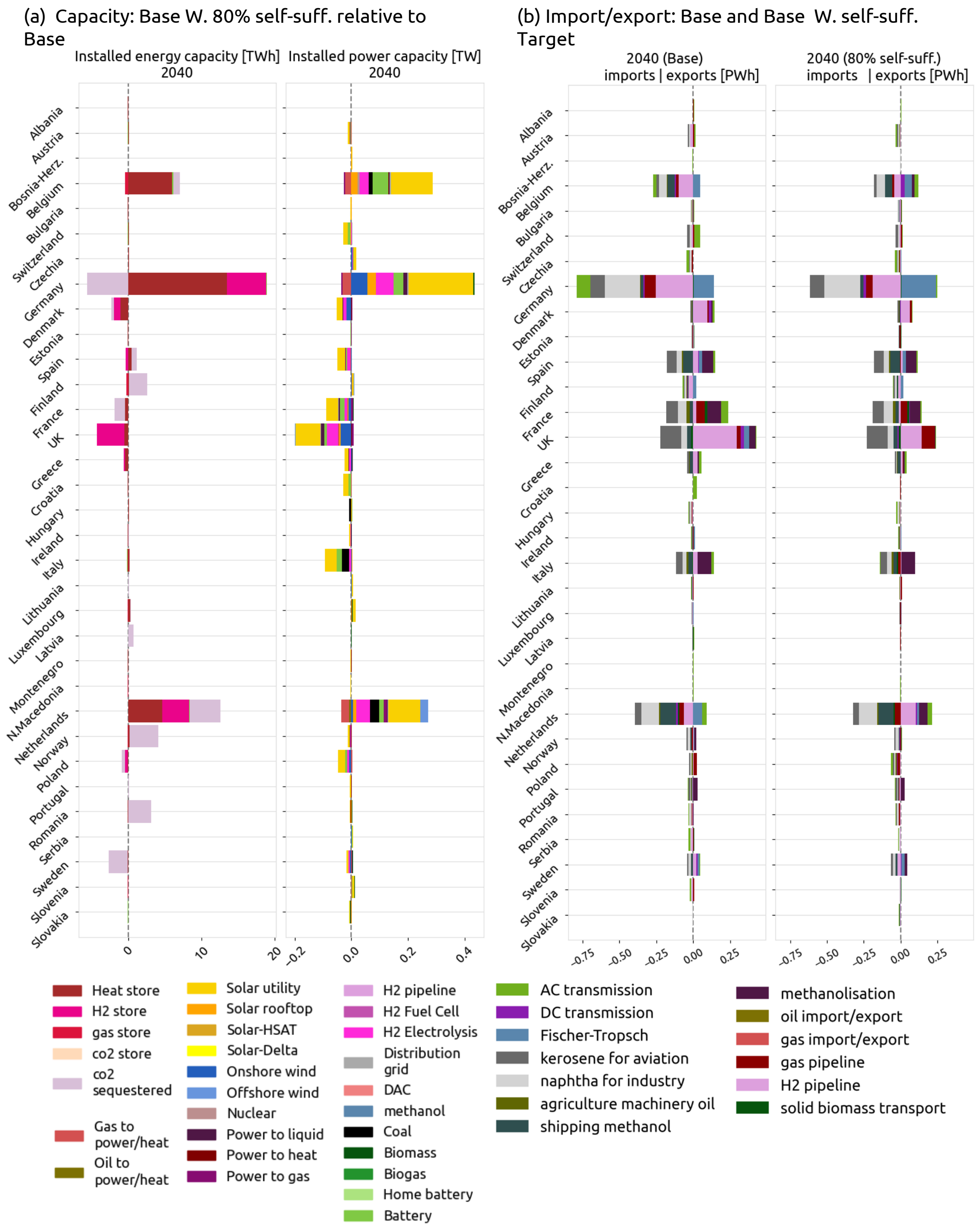

Supplementary Fig. 21: Effect of 80% self-sufficiency for Base scenarios. (a) Changes in the total installed capacity of different technologies for the base scenario with 80% self-sufficiency target relative to the base scenario in 2040. (b) changes in import and export of different countries for the base scenario and base scenario with 80% self-sufficiency target in 2040. Installed energy capacity is shown for storage technologies and power capacity for the rest.

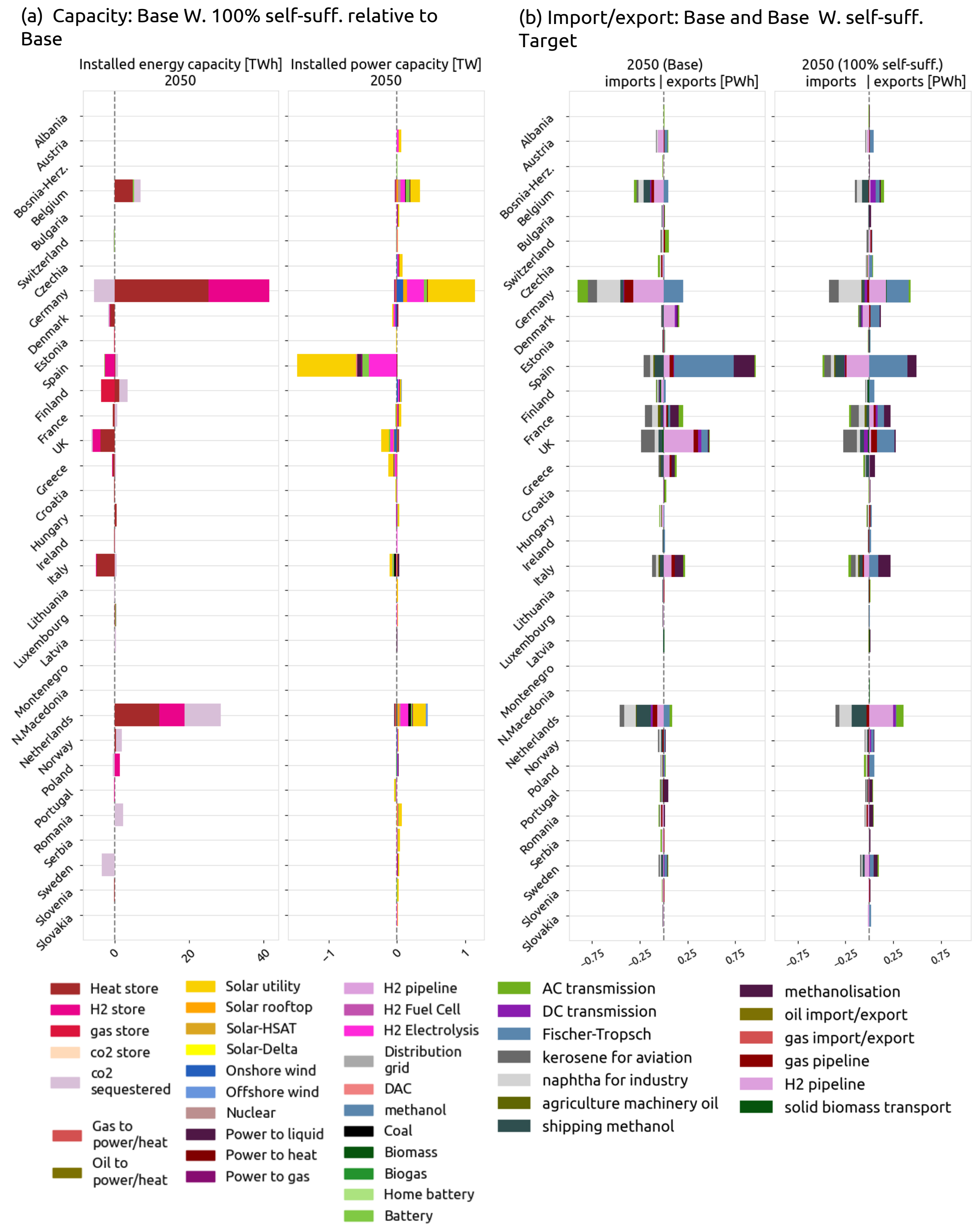

Supplementary Fig. 22: Effect of 100% self-sufficiency for Base scenarios. (a) Changes in the total installed capacity of different technologies for the base scenario with 100% self-sufficiency target relative to the base scenario in 2050. (b) Changes in import and export of different countries for the base scenario and base scenario with 100% self-sufficiency target in 2050.Installed energy capacity is shown for storage technologies and power capacity for the rest.

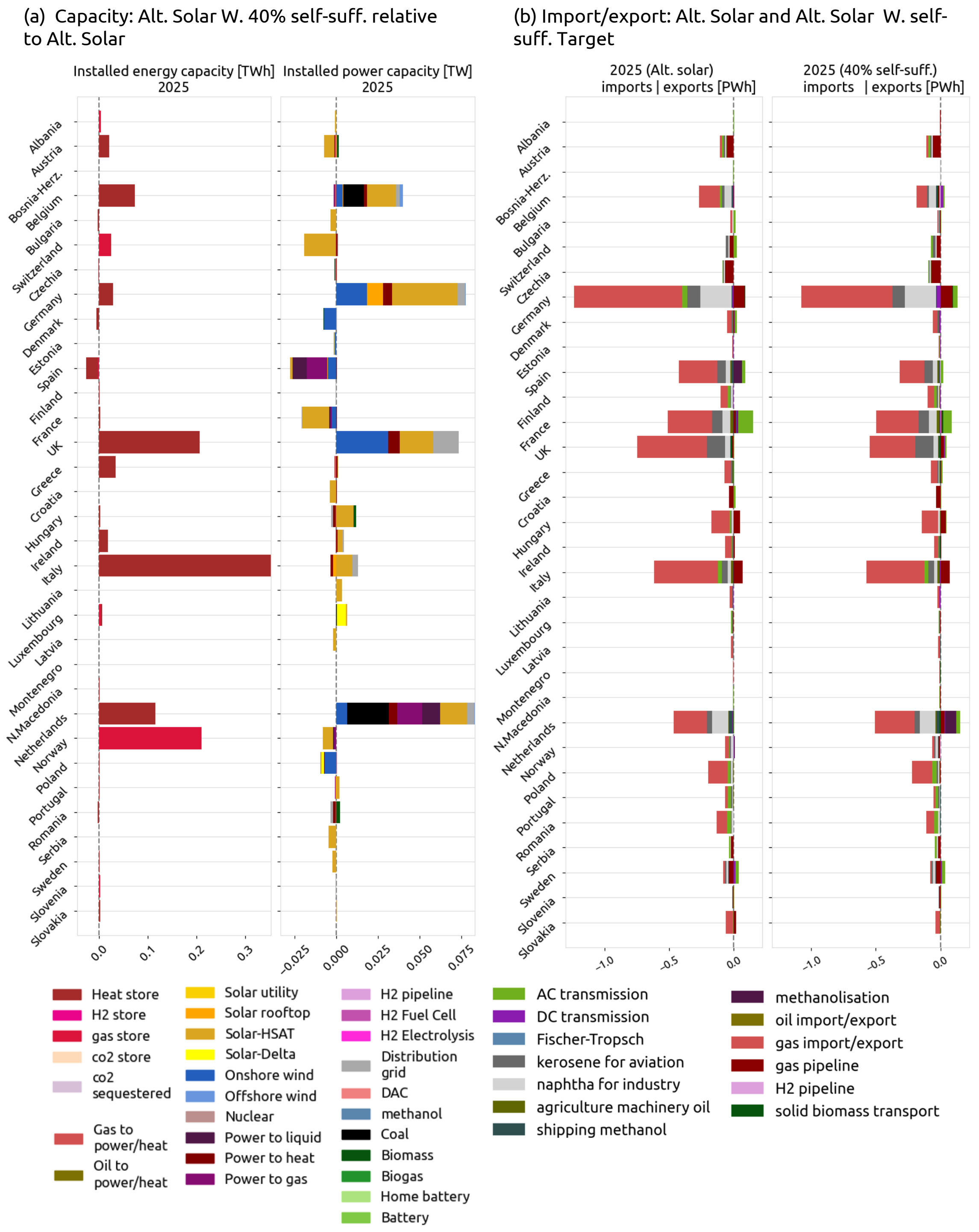

Supplementary Fig. 23: Effect of 40% self-sufficiency for scenarios with alternative solar configurations. (a) Changes in the installed capacity of different technologies for the scenario with alternative solar configurations and 40% self-sufficiency target relative to the scenario with alternative solar configurations in 2025. (b) Changes in import and export of different countries for the scenario with alternative solar configurations and the scenario with alternative solar configurations plus 40% self-sufficiency target in 2025. Installed energy capacity is shown for storage technologies and power capacity for the rest.

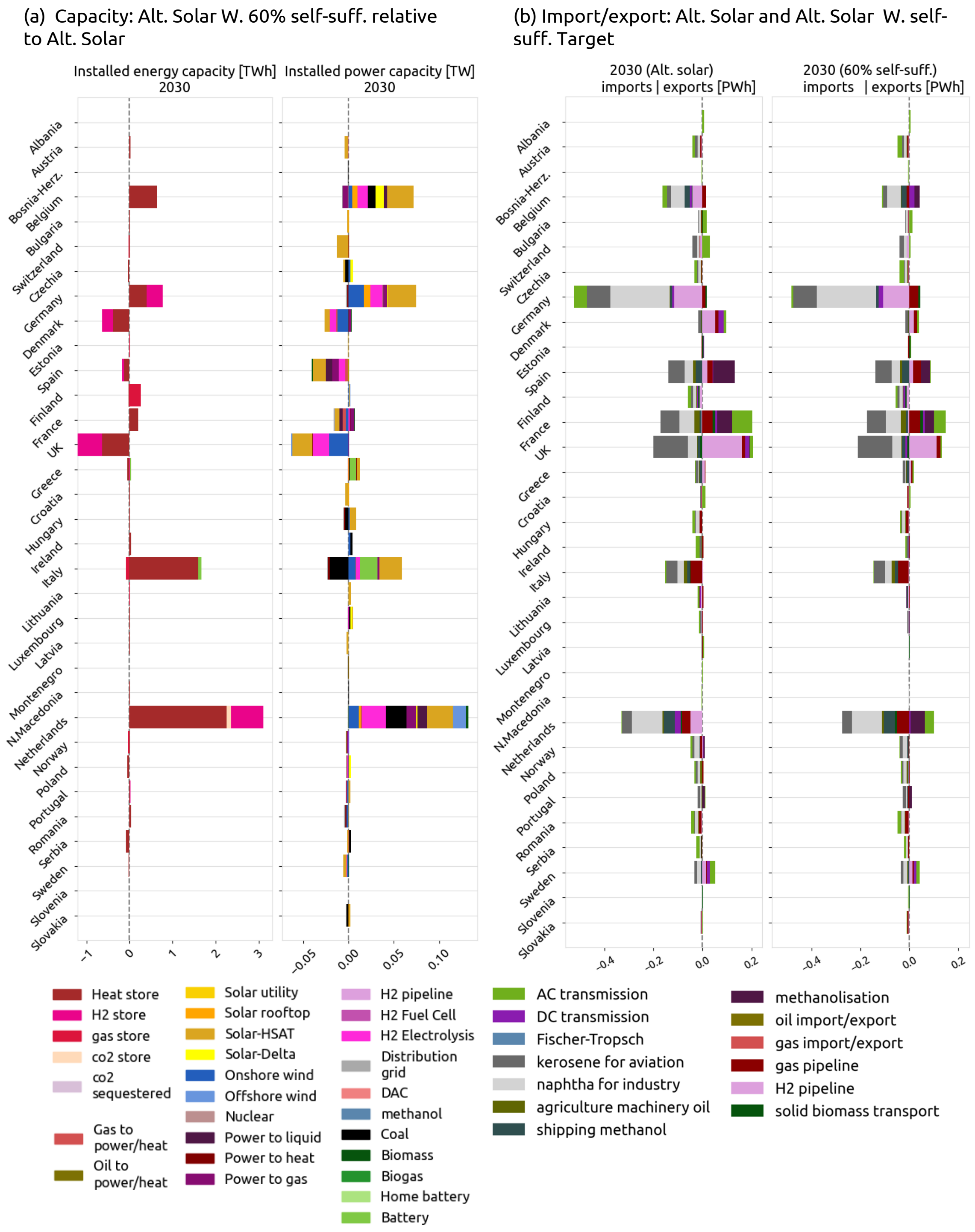

Supplementary Fig. 24: Effect of 60% self-sufficiency for scenarios with alternative solar configurations. (a) Changes in the installed capacity of different technologies for the scenario with alternative solar configurations and 60% self-sufficiency target relative to the scenario with alternative solar configurations in 2030. (b) Changes in import and export of different countries for the scenario with alternative solar configurations and the scenario with alternative solar configurations plus 60% self-sufficiency target in 2030. Installed energy capacity is shown for storage technologies and power capacity for the rest.

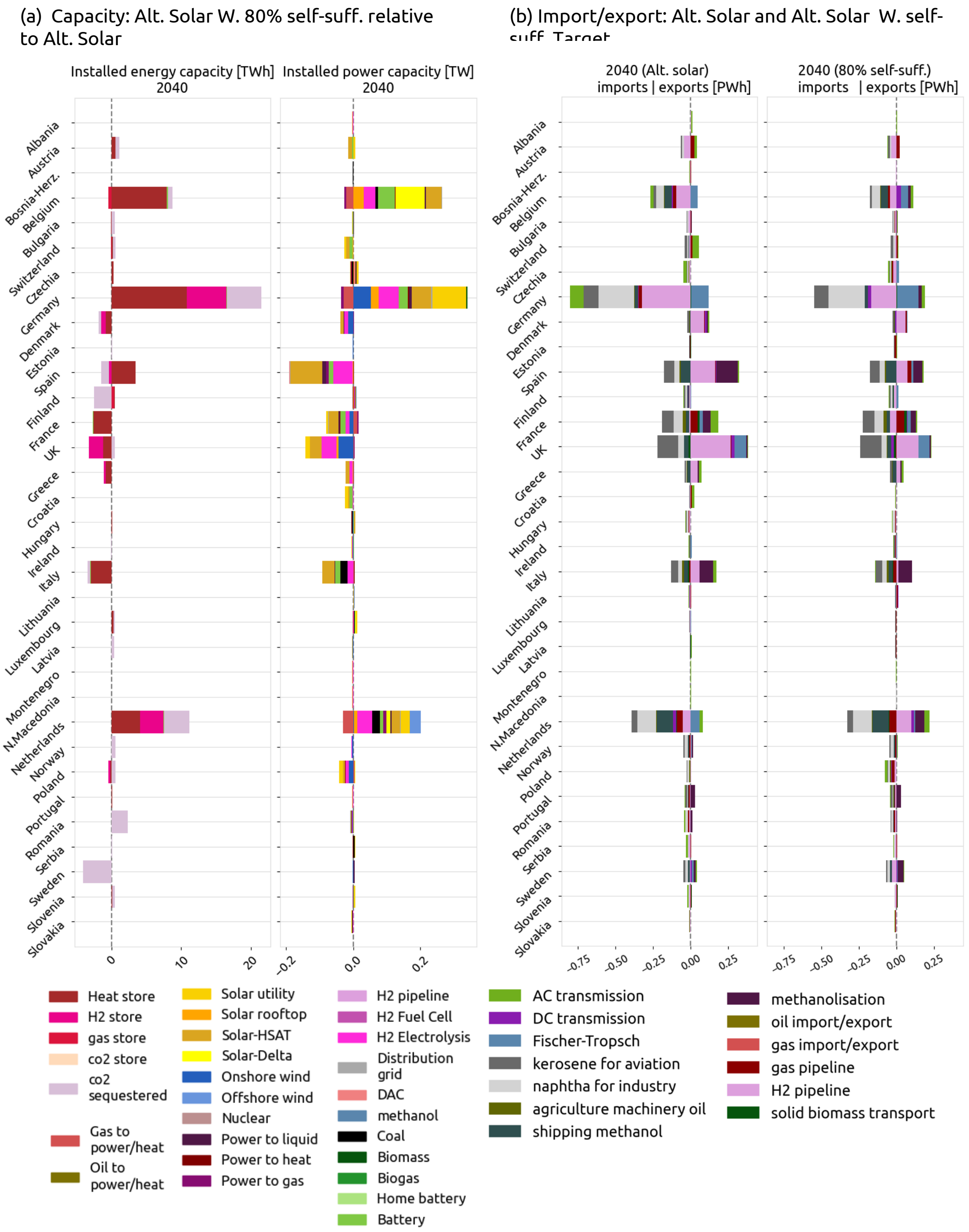

Supplementary Fig. 25: Effect of 80% self-sufficiency for scenarios with alternative solar configurations. (a) Changes in the installed capacity of different technologies for the scenario with alternative solar configurations and 80% self-sufficiency target relative to the scenario with alternative solar configurations in 2040. (b) Changes in import and export of different countries for the scenario with alternative solar configurations and the scenario with alternative solar configurations plus 80% self-sufficiency target in 2040. Installed energy capacity is shown for storage technologies and power capacity for the rest.

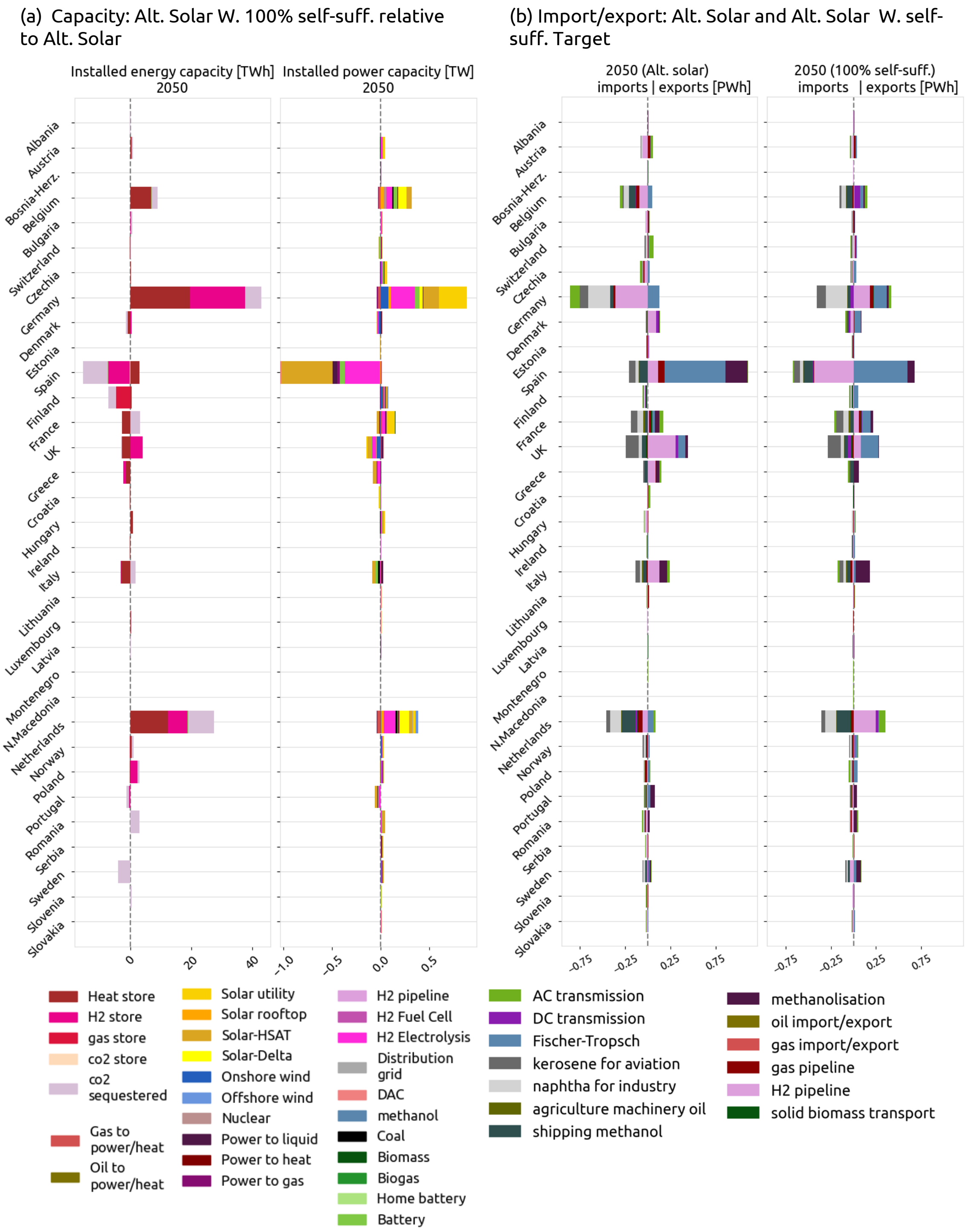

Supplementary Fig. 26: Effect of 100% self-sufficiency for scenarios with alternative solar configurations. (a) Changes in the installed capacity of different technologies for the scenario with alternative solar configurations and 100% self-sufficiency target relative to the scenario with alternative solar configurations in 2050, and (b) Changes in import and export of different countries for the scenario with alternative solar configurations and the scenario with alternative solar configurations plus 100% self-sufficiency target in 2050. Installed energy capacity is shown for storage technologies and power capacity for the rest.

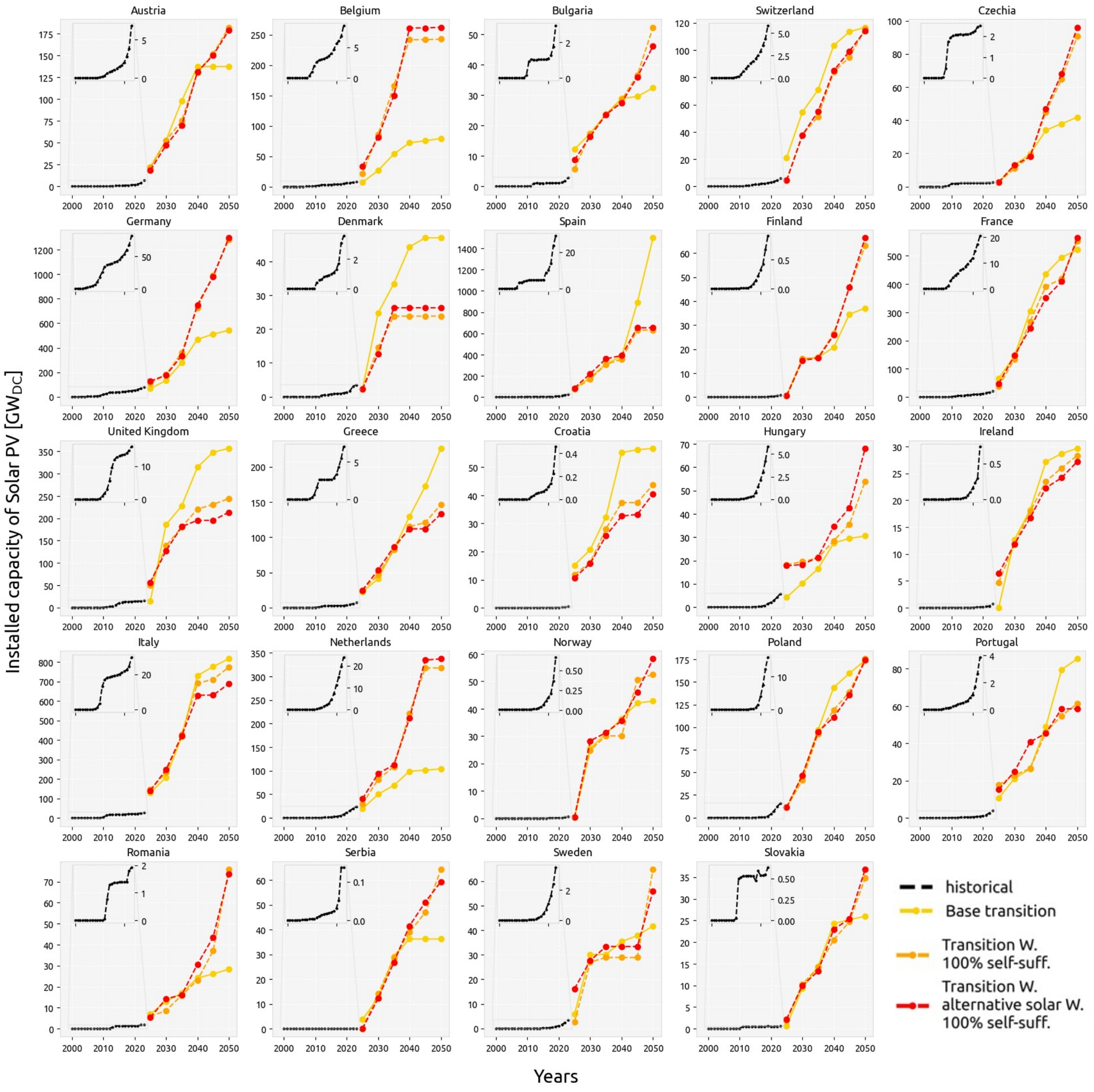

Supplementary Fig. 27: Historical (data from IRENA [26]) and future cumulative installed capacity of solar PV for different European countries. Each plot shows the results for the base transition, transition with a 100% self-sufficiency target, and transition with selected alternative solar configurations under a 100% self-sufficiency target.

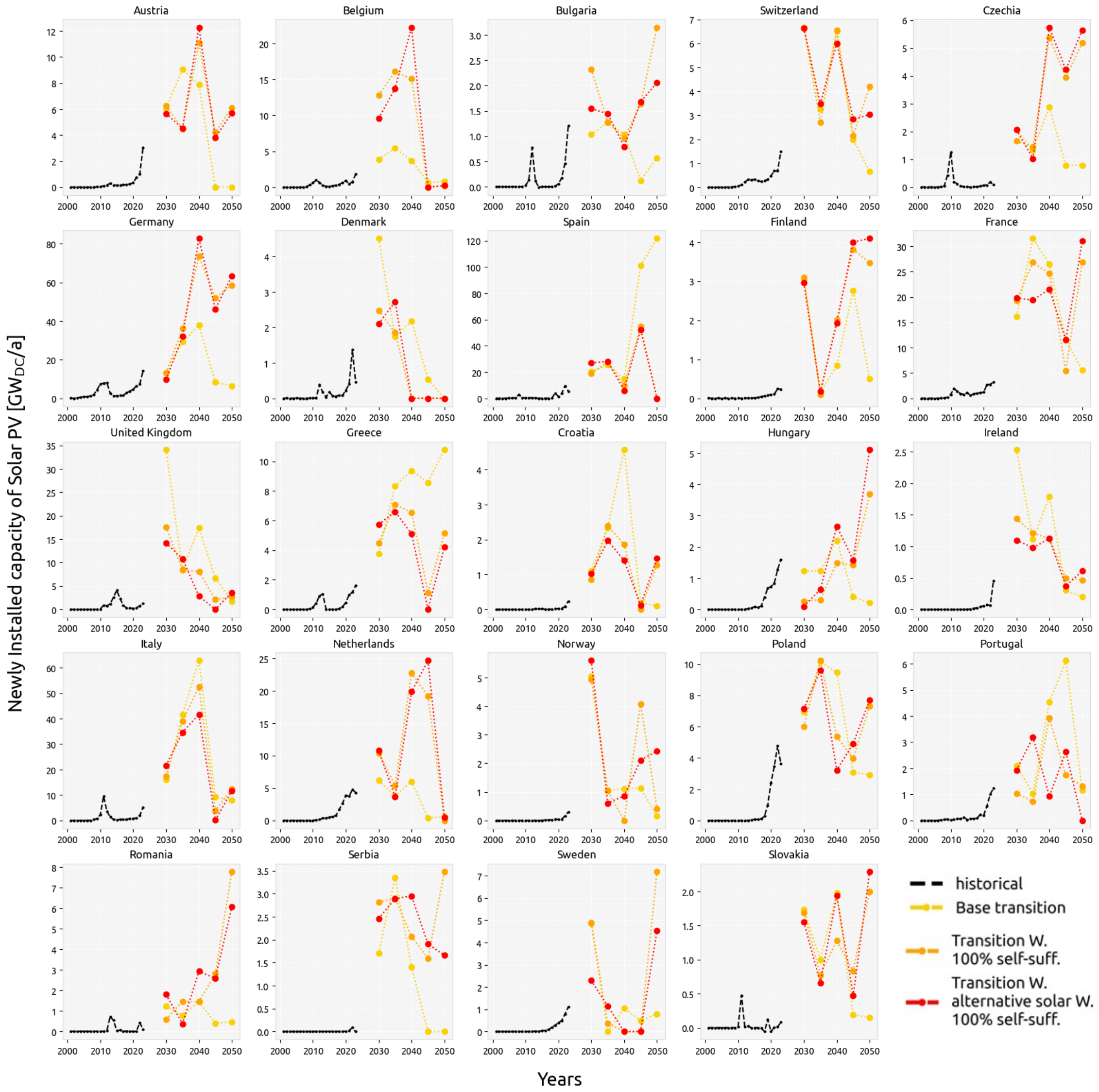

Supplementary Fig. 28: Historical (data from IRENA [26]) and future newly installed capacity of solar PV for different European countries. Each plot shows the results for the base transition, transition with a 100% self-sufficiency target, and transition with selected alternative solar configurations under a 100% self-sufficiency target.

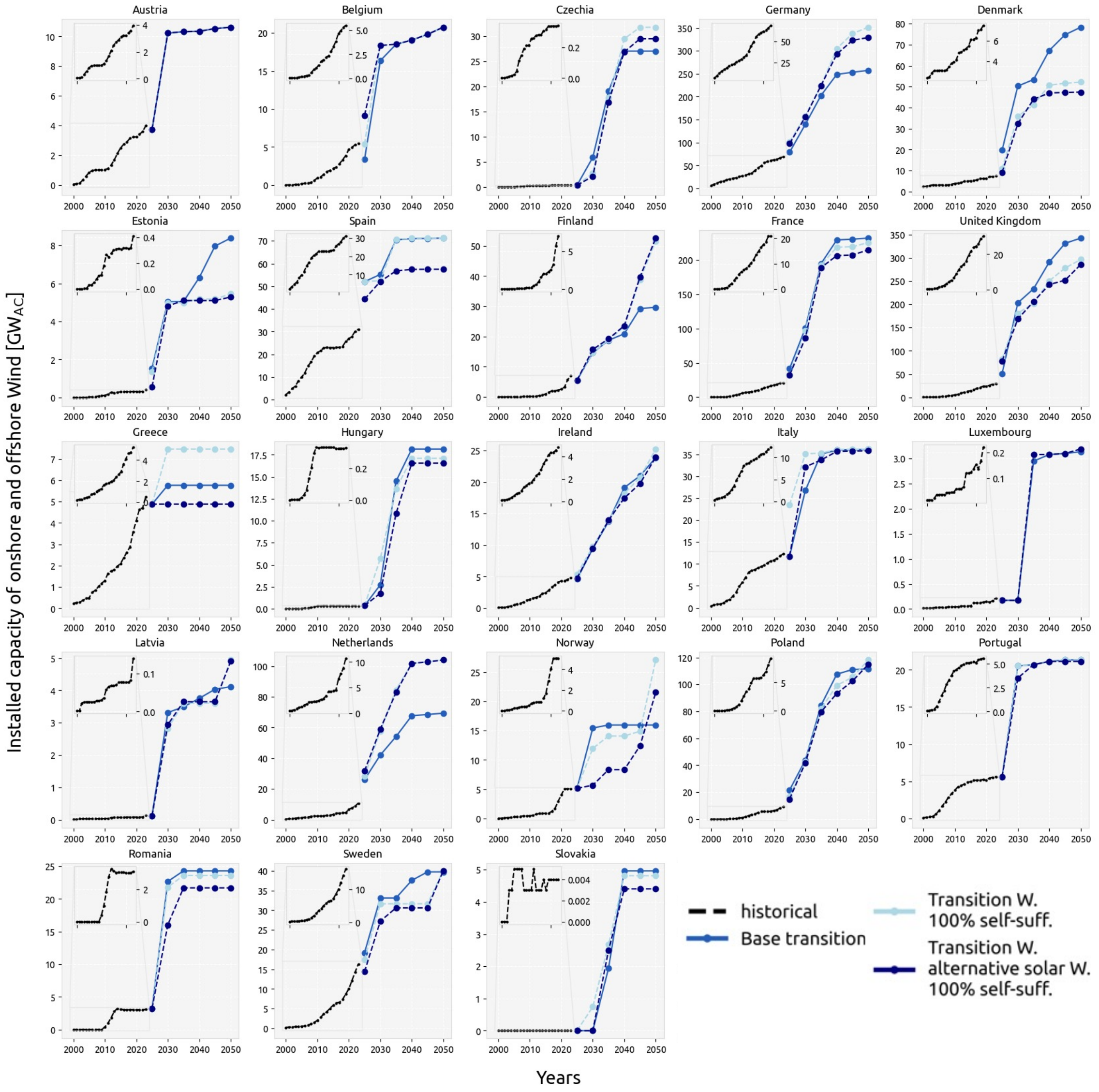

Supplementary Fig. 29: Historical (data from IRENA [26]) and future cumulative installed capacity of onshore and offshore wind for different European countries. Each plot shows the results for the base transition, transition with a 100% self-sufficiency target, and transition with selected alternative solar configurations under a 100% self-sufficiency target.

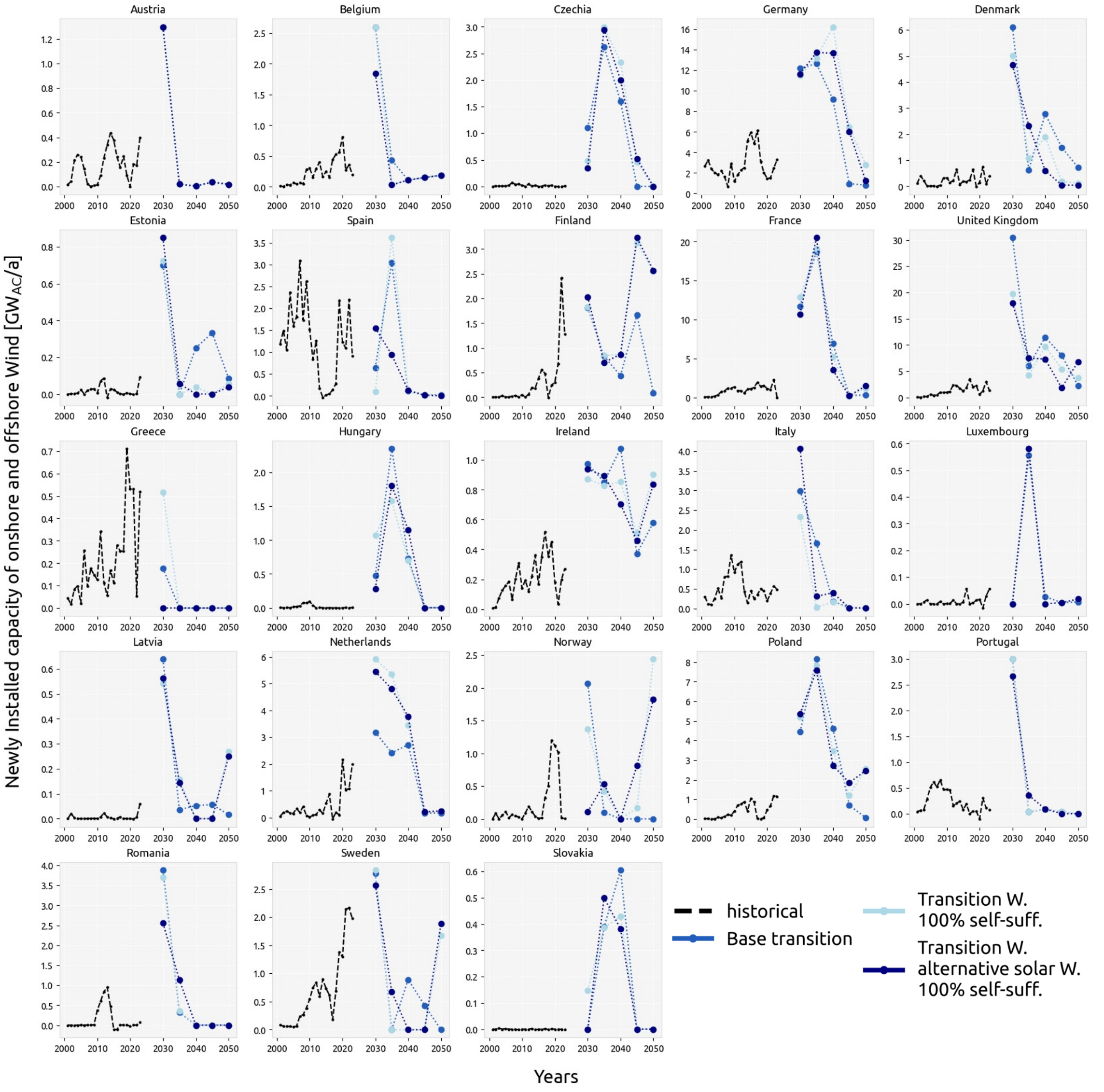

Supplementary Fig. 30: Historical (data from IRENA [26]) and future newly installed capacity of onshore and offshore wind for different European countries. Each plot shows the results for the base transition, transition with a 100% self-sufficiency target, and transition with selected alternative solar configurations under a 100% self-sufficiency target.

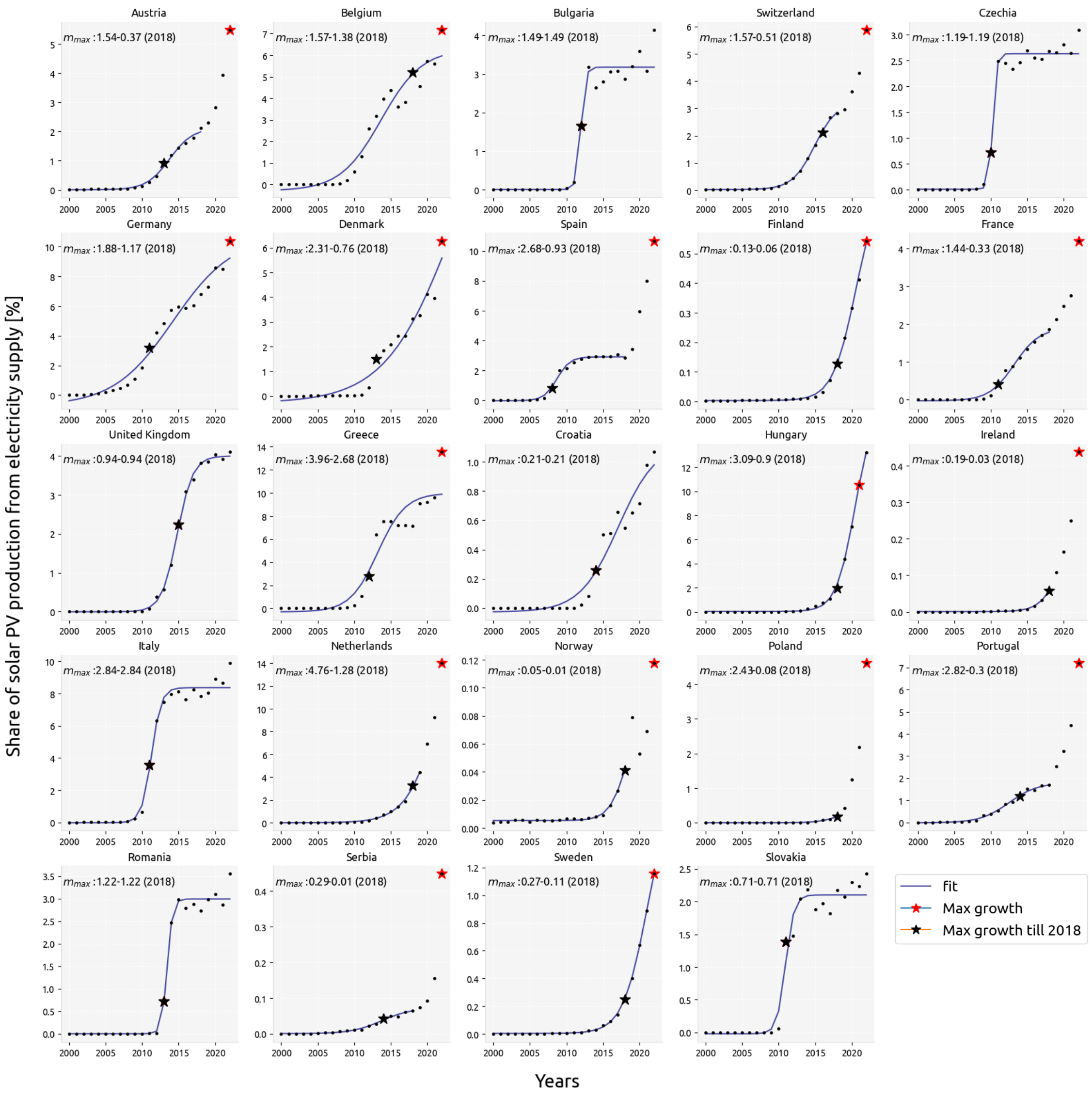

Supplementary Fig. 31: Historical (data from IRENA[26]) share of solar PV generation from total electricity generation for different European countries. The blue line shows a fit for the data produced with the logistic function, which has a characteristic S-shaped curve, using the SciPy package (This function is also referred to as the logistic model in the work of Cherp at al.[27]). Since many countries already show a second ramp-up period after the tail of the S-curve, a fit cannot be produced for all the data, in which case the fit is produced only for data up to 2018 to match the study by Cherp and co-authors. The black star indicates the maximum growth rate (m_{max}) until 2018 and the red star indicates the same for all the data, with both numbers displayed on each figure. Austria, Belgium, Germany, Spain, and Netherlands are clear examples of countries where the highest growth has taken place very recently (2023), with the value up to five times higher than the maximum growth before 2019.

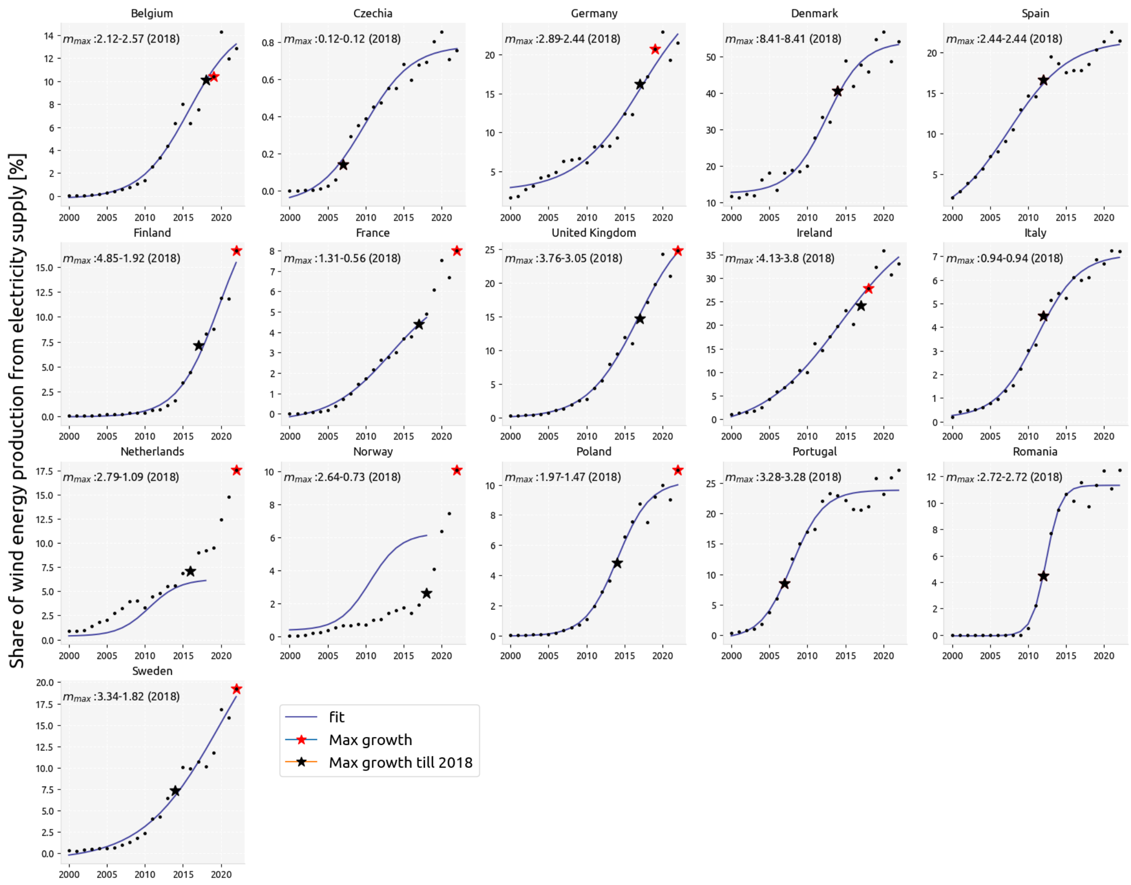

Supplementary Fig. 32: Historical (data from IRENA [26]) share of wind generation (both onshore and offshore) from total electricity generation for different European countries. The blue line shows a fit for the data produced with the logistic function, which has a characteristic S-shaped curve, using the SciPy package. Since some countries already show a second ramp-up period after the tail of the S-curve, a fit cannot be produced for all the data, in which case the fit is produced only for data up to 2018. The black star indicates the maximum growth rate (m_{max}) until 2018 and the red star indicates the same for all the data, with both numbers displayed on each figure. Germany, France, and Netherlands are clear examples of countries where the highest growth has taken place very recently (2023), with the value up to 3.5 times higher than the maximum growth before 2019.

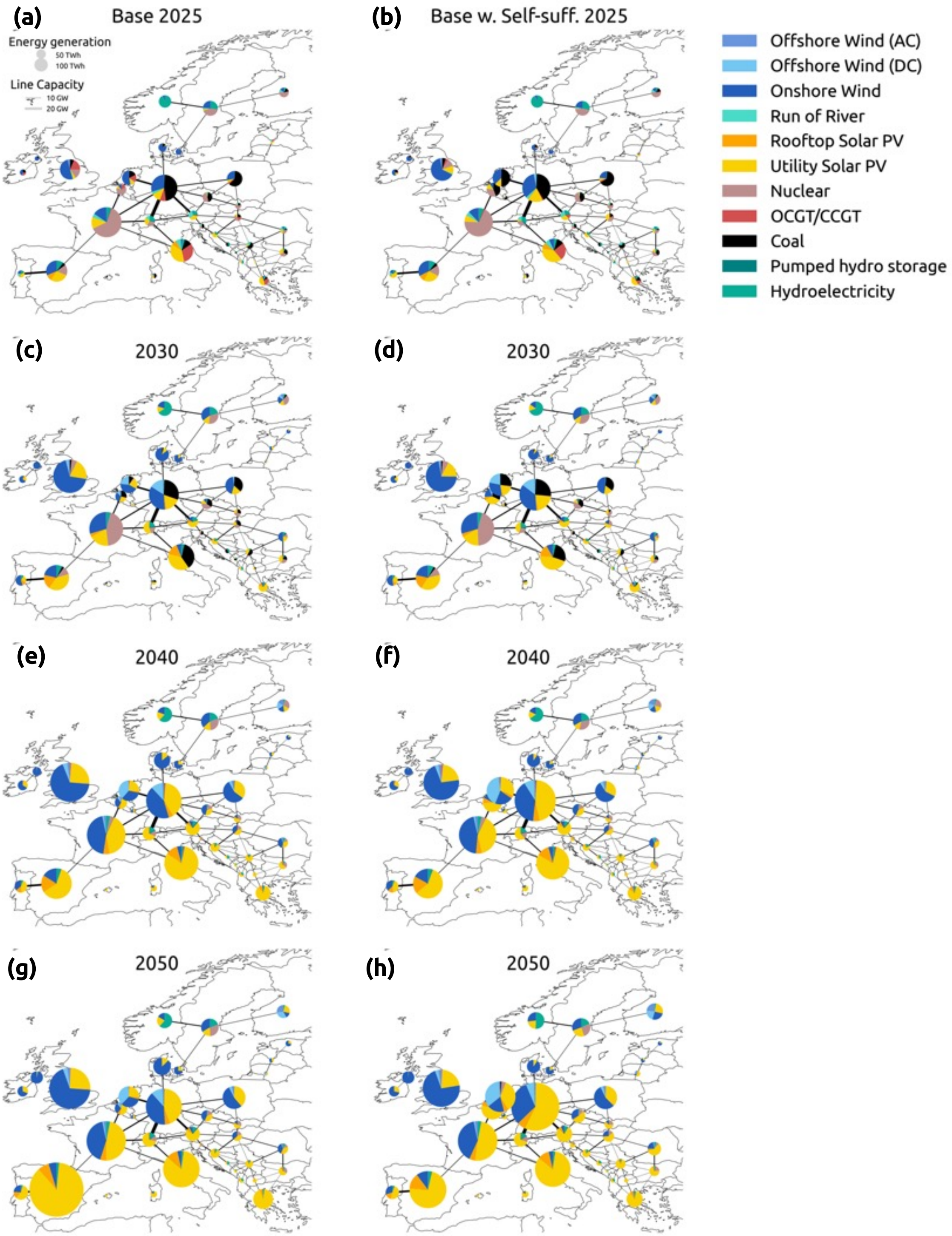

Supplementary Fig. 33: Energy generation map for the base scenarios. (a,c,e,g) Without and (b,d,f,h) with self-sufficiency targets during the transition showing the share of major technologies in total annual electricity generation.

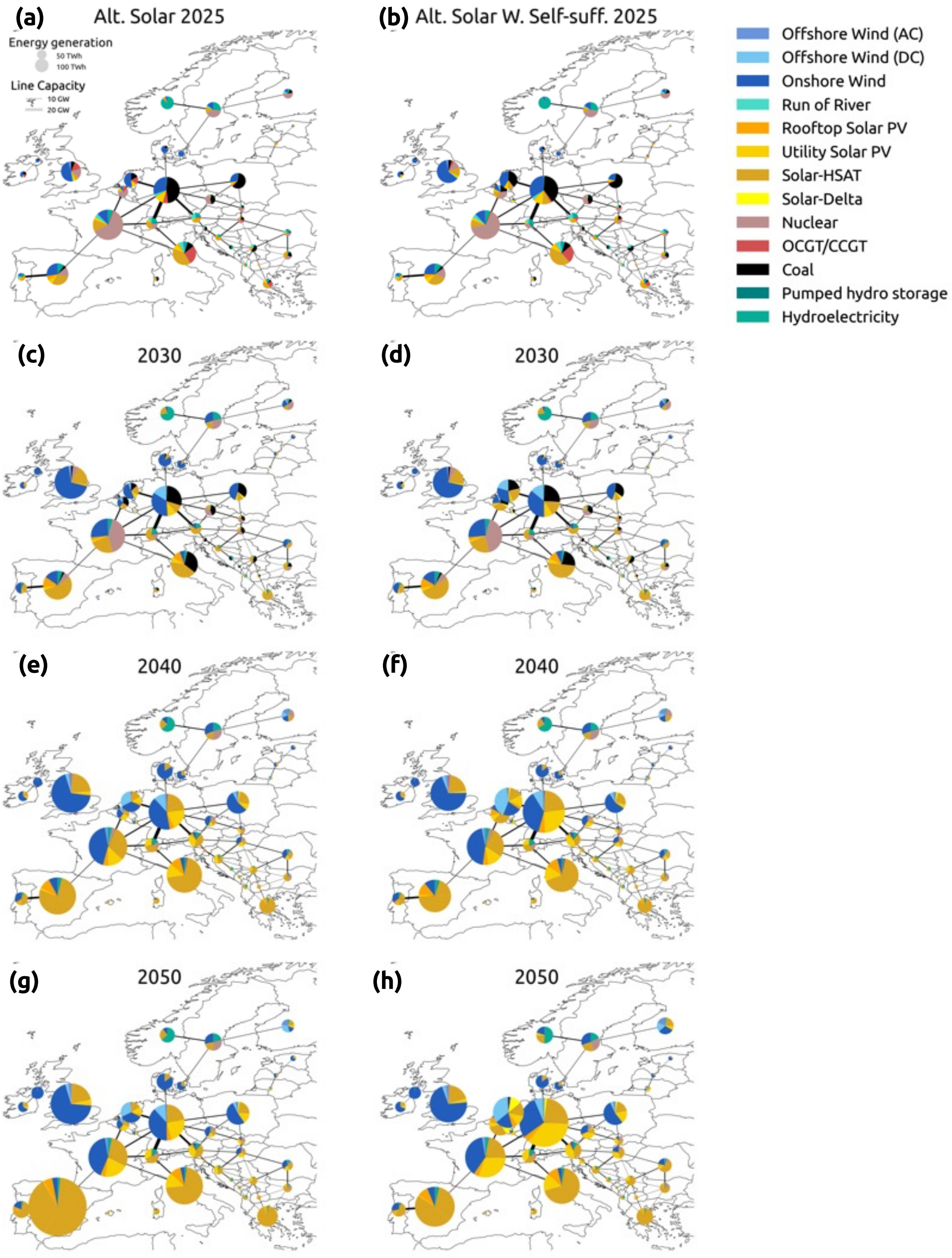

Supplementary Fig. 34: Energy generation map for the scenarios with alternative solar configurations. (a,c,e,g) Without and (b,d,f,h) with self-sufficiency targets during the transition showing the share of major technologies in total annual electricity generation

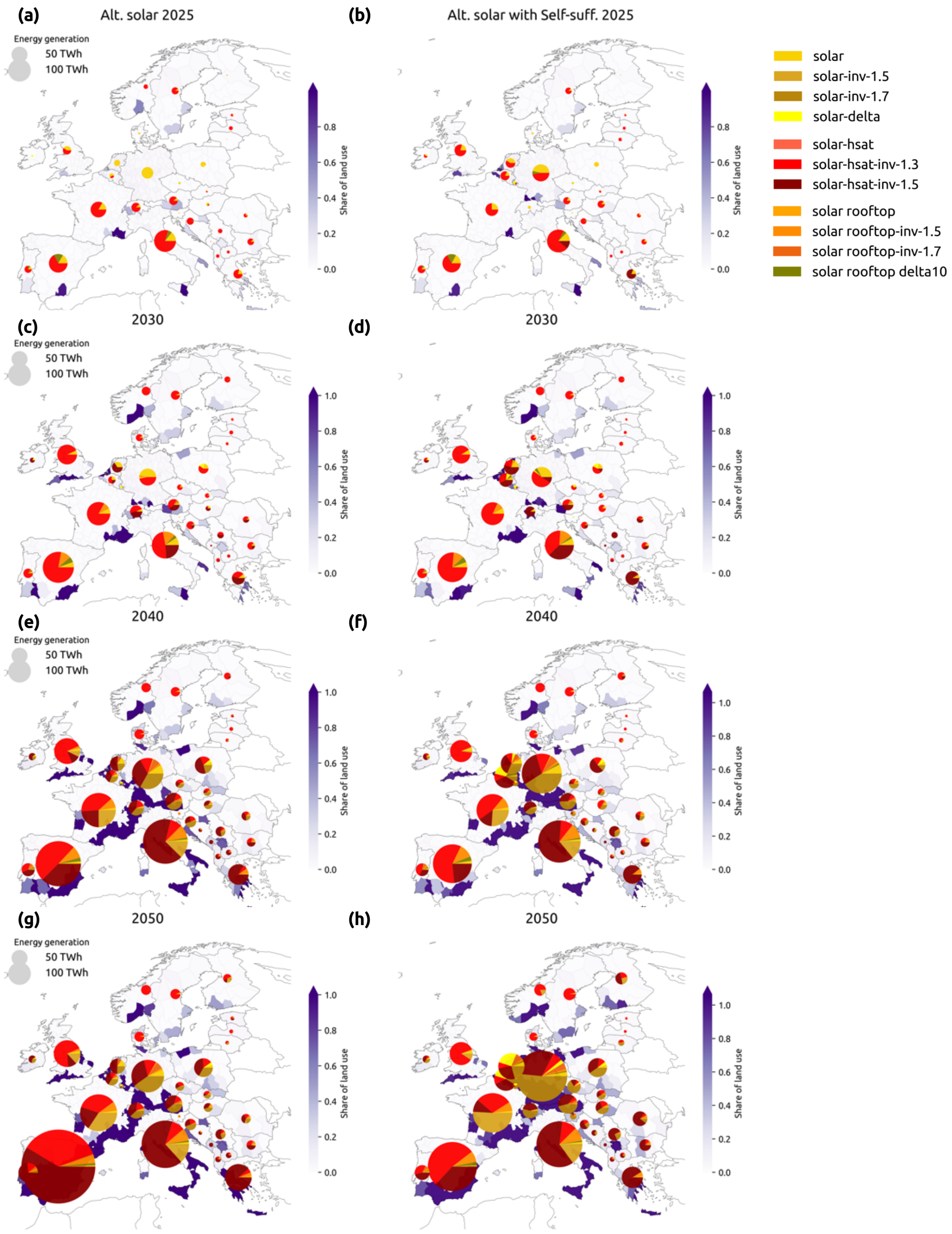

Supplementary Fig. 35: Solar generation map by configuration and regional cumulative land-use of solar PV technologies for year years 2025, 2030, and 2040. (a,c,e,g) Transition with selected alternative solar configurations. (b,d,f,h) Transition with selected alternative solar configurations with self-sufficiency target.

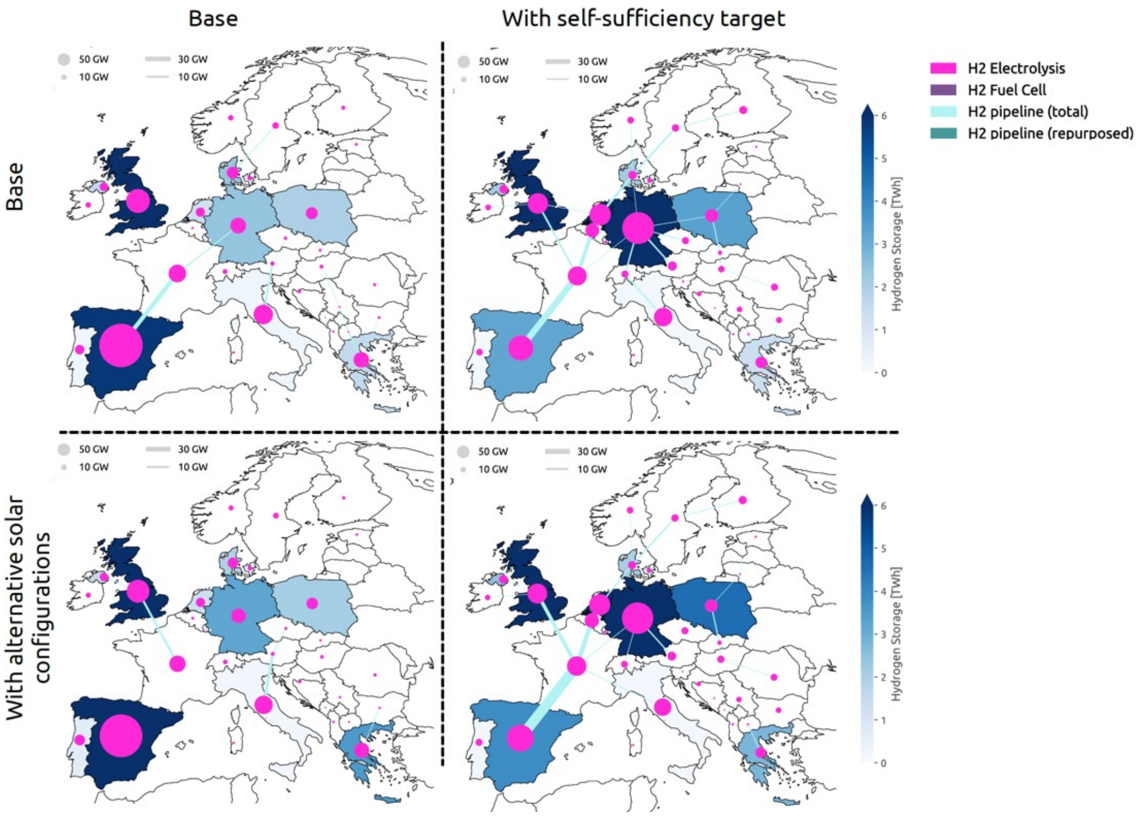

Supplementary Fig. 36: Map of energy infrastructure for hydrogen generation and transport for different scenarios in 2050. Addition of alternative solar configurations does not impact the infrastructure layout noticeably. The self-sufficiency requirement causes a large shift of installed capacities from Spain to Germany, Belgium, and Netherlands as they increase their hydrogen production.

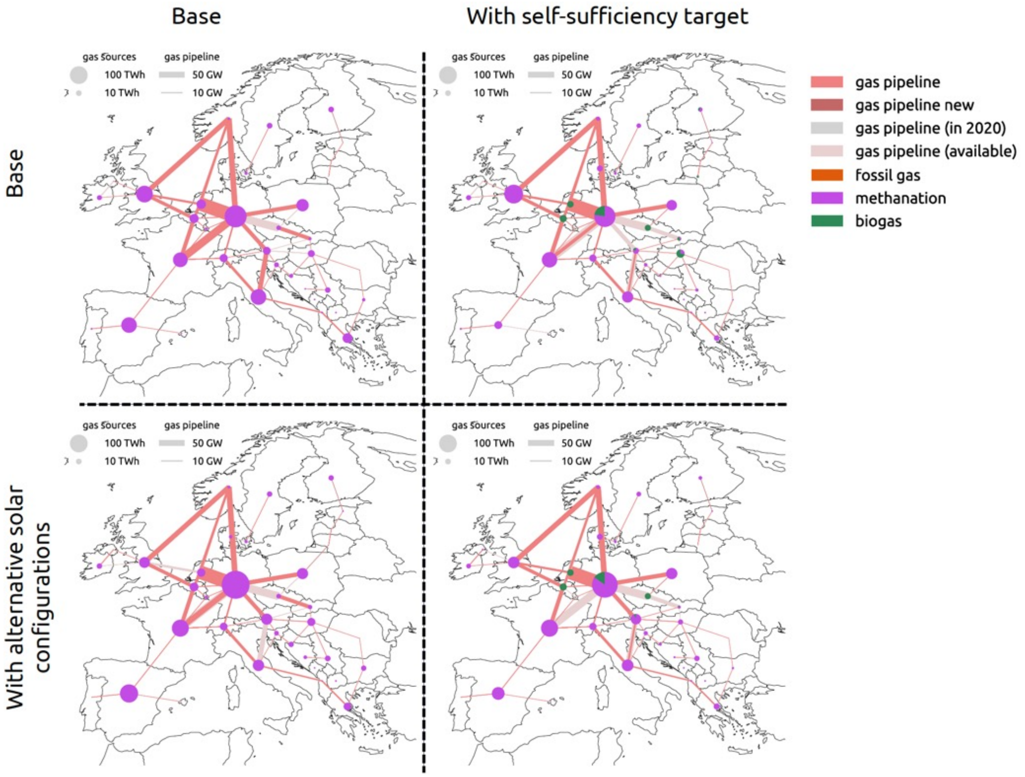

Supplementary Fig. 37: Map of energy infrastructure for methane generation and transport for different scenarios in 2050. Addition of alternative solar configurations does not impact the infrastructure layout noticeably. The self-sufficiency requirement triggers the installation of biomass plants in Germany, Netherlands, and Belgium, as they reduce their import of hydrogen that is required for methanation (see Supplementary Fig. 22).

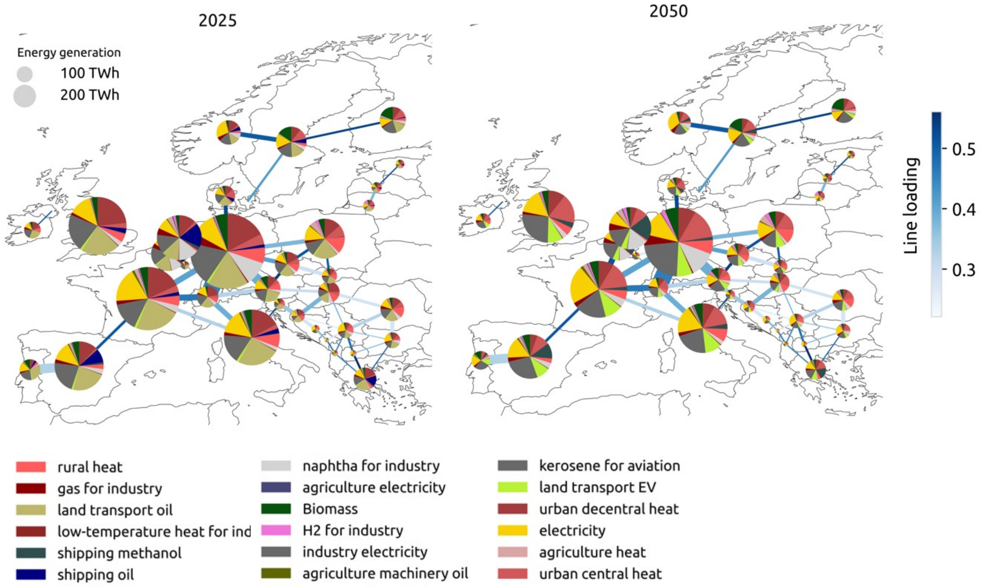

Supplementary Fig. 38: Total energy demand by sector for years 2025 and 2050. Notice the transition of shipping fuel from oil to methanol and land transport from oil to electricity.

Supplementary Tables

The table below presents projected costs for 2030, with the complete set of technology data assumptions for all investment periods available in the Zenodo repository of this study.

Please note that the cost data utilized in PyPSA-Eur (accessible at PyPSA/Technology-data) are continuously updated to reflect the latest datasets and inflation rate information. Consequently, slight variations may exist between the table below and earlier or future versions of the online repository. The costs presented here correspond to Technology data version v0.8.1. As noted in Supplementary Note 3, the solar PV costs in this study are lower due to the implementation of inverter dimensioning.

Supplementary Table 4: Summary of the main technology assumptions.

**Technology**	**Parameter**	**Value**	**Unit**	**Currency year**
Solar-utility	AC share of investment	21.6624	%	2020
	FOM	2.4757	%/year	2020
	investment	320.8874	EUR/kW_e	2020
	lifetime	40	years	2020
Solar-HSAT	AC share of investment	18.4244	%	2020
	FOM	2.2884	%/year	2020
	investment	377.4862	EUR/kW_e	2020
	lifetime	40	years	2020
Solar-rooftop	FOM	1.4234	%/year	2020
	discount rate	0.04	per unit	2015
	investment	668.0004	EUR/kW_e	2020
	lifetime	40	years	2020
Onshore wind	FOM	1.2167	%/year	2015
	VOM	1.4286	EUR/MWh	2015
	investment	1095.8533	EUR/kW	2015
	lifetime	30	years	2015
Offshore wind	FOM	2.3185	%/year	2020
	VOM	0.0212	EUR/MWhel	2015
	investment	1682.1226	EUR/kW_e, 2020	2020
	lifetime	30	years	2020
Nuclear	FOM	1.27	%/year	2023
	VOM	3.5464	EUR/MWh_e	2023
	efficiency	0.326	p.u.	2023
	fuel	3.4122	EUR/MWh_th	2010
	investment	8594.1354	EUR/kW_e	2023
	lifetime	40	years	2023
Open-cycle gas turbine (OCGT)	FOM	1.7795	%/year	2015
	VOM	4.762	EUR/MWh	2015
	efficiency	0.41	per unit	2015
	investment	460.5804	EUR/kW	2015
	lifetime	25	years	2015
Closed-cycle gas turbine (CCGT)	FOM	3.3494	%/year	2015
	VOM	4.4445	EUR/MWh	2015
	c_b	2	50°C/100°C	2015
	c_v	0.15	50°C/100°C	2015
	efficiency	0.58	per unit	2015

Continued on next page

Supplementary Table 4: Summary of the main technology assumptions. (Continued)

Technology	Parameter	Value	Unit	Currency year
	investment	878.324	EUR/kW	2015
	lifetime	25	years	2015
Pumped hydro storage	FOM	1	%/year	2015
	efficiency	0.75	per unit	2015
	investment	2274.8177	EUR/kWel	2010
	lifetime	80	years	2015
Hydroelectricity	FOM	1	%/year	2015
	efficiency	0.9	per unit	2015
	investment	2274.8177	EUR/kWel	2010
	lifetime	80	years	2015
Run of river	FOM	2	%/year	2015
	efficiency	0.9	per unit	2015
	investment	3412.2266	EUR/kWel	2010
	lifetime	80	years	2015
Coal	CO_2 intensity	0.3361	tCO_2/MWh_th	
	FOM	1.31	%/year	2023
	VOM	3.2612	EUR/MWh_e	2023
	efficiency	0.33	p.u.	2023
	fuel	9.5542	EUR/MWh_th	2010
	investment	3827.1629	EUR/kW_e	2023
	lifetime	40	years	2023
Oil	CO_2 intensity	0.2571	tCO_2/MWh_th	
	FOM	2.463	%/year	2015
	VOM	6.3493	EUR/MWh	2015
	efficiency	0.35	per unit	2015
	fuel	52.9111	EUR/MWhth	2015
	investment	362.97	EUR/kW	2015
	lifetime	25	years	2015
Electrolysis	FOM	4	%/year	2020
	efficiency	0.6217	per unit	2020
	efficiency-heat	0.2228	per unit	2020
	investment	550	EUR/kW_e	2020
	lifetime	25	years	2020
Fuel cell	FOM	5	%/year	2015
	c_b	1.25	50°C/100°C	2015
	efficiency	0.5	per unit	2015
	investment	1164.0438	EUR/kW_e	2015
	lifetime	10	years	2015
Battery inverter	FOM	0.3375	%/year	2015
	efficiency	0.96	per unit	2015
	investment	169.3155	EUR/kW	2015
	lifetime	10	years	2015
Home battery inverter	FOM	0.3375	%/year	2015
	efficiency	0.96	per unit	2015
	investment	241.3377	EUR/kW	2015
	lifetime	10	years	2015

Continued on next page

Supplementary Table 4: Summary of the main technology assumptions. (Continued)

**Technology**	**Parameter**	**Value**	**Unit**	**Currency year**
Battery storage	investment	150.2675	EUR/kWh	2015
	lifetime	25	years	2015
Home battery storage	investment	214.7158	EUR/kWh	2015
	lifetime	25	years	2015
Direct air capture	FOM	4.95	%/year	2015
	compression-electricity-input	0.15	MWh/tCO_2	2015
	compression-heat-output	0.2	MWh/tCO_2	2015
	electricity-input	0.4	MWh_el/t_CO_2	2015
	heat-input	1.6	MWh_th/t_CO_2	2015
	heat-output	1	MWh/tCO_2	2015
	investment	6349329.76	EUR/(tCO_2/h)	2015
	lifetime	20	years	2015
Biomass	FOM	4.5269	%/year	2015
	efficiency	0.468	per unit	2015
	fuel	7.4076	EUR/MWhth	2015
	investment	2337.6116	EUR/kWel	2015
	lifetime	30	years	2015
Biomass CHP	FOM	3.5822	%/year	2015
	VOM	2.222	EUR/MWh_e	2015
	c_b	0.4564	40°C/80°C	2015
	c_v	1	40°C/80°C	2015
	efficiency	0.3003	per unit	2015
	efficiency-heat	0.7083	per unit	2015
	investment	3397.1862	EUR/kW_e	2015
	lifetime	25	years	2015
Biomass boiler	efficiency	0.86	per unit	2015
	investment	687.1015	EUR/kW_th	2015
	lifetime	20	years	2015
	pelletizing cost	9	EUR/MWh_pellets	2019
Central air-sourced heat pump	FOM	0.2336	%/year	2015
	VOM	2.6561	EUR/MWh_th	2015
	efficiency	3.6	per unit	2015
	investment	906.0988	EUR/kW_th	2015
	lifetime	25	years	2015
Central gas CHP	FOM	3.3214	%/year	2015
	VOM	4.4445	EUR/MWh	2015
	c_b	1	50°C/100°C	2015
	c_v	0.17	per unit	2015
	efficiency	0.41	per unit	2015
	investment	592.6041	EUR/kW	2015
	lifetime	25	years	2015
	p_nom_ratio	1	per unit	2015
Central gas boiler	FOM	3.8	%/year	2015
	VOM	1.0582	EUR/MWh_th	2015
	efficiency	1.04	per unit	2015
	investment	52.9111	EUR/kW_th	2015
	lifetime	25	years	2015
Central solar thermal	FOM	1.4	%/year	2015
	investment	148151.028	EUR/1000m2	2015
	lifetime	20	years	2015

Continued on next page

Supplementary Table 4: Summary of the main technology assumptions. (Continued)

**Technology**	**Parameter**	**Value**	**Unit**	**Currency year**
Central ground-sourced heat pump	FOM	0.394	%/year	2015
	VOM	1.3268	EUR/MWh_th	2015
	efficiency	1.73	per unit	2015
	investment	537.1533	EUR/kW_th excluding drive energy	2015
	lifetime	25	years	2015
Central resistive heater	FOM	1.7	%/year	2015
	VOM	1.0582	EUR/MWh_th	2015
	efficiency	0.99	per unit	2015
	investment	63.4933	EUR/kW_th	2015
	lifetime	20	years	2015
Central solid biomass CHP	FOM	2.8661	%/year	2015
	VOM	4.8512	EUR/MWh_e	2015
	c_b	0.3506	50°C/100°C	2015
	c_v	1	50°C/100°C	2015
	efficiency	0.2699	per unit	2015
	efficiency-heat	0.8245	per unit	2015
	investment	3544.5017	EUR/kW_e	2015
	lifetime	25	years	2015
	p_nom_ratio	1	per unit	2015
Waste CHP	FOM	2.355	%/year	2015
	VOM	28.064	EUR/MWh_e	2015
	c_b	0.2918	50°C/100°C	2015
	c_v	1	50°C/100°C	2015
	efficiency	0.2081	per unit	2015
	efficiency-heat	0.7619	per unit	2015
	investment	8582.5944	EUR/kW_e	2015
	lifetime	25	years	2015
Central water tank storage	FOM	0.551	%/year	2015
	investment	0.5761	EUR/kWhCapacity	2015
	lifetime	25	years	2015
Decentral water tank storage	FOM	1	%/year	2015
	discount rate	0.04	per unit	2015
	investment	19.446	EUR/kWh	2015
	lifetime	20	years	2015
Fischer-Tropsch	FOM	3	%/year	2017
	VOM	4.7263	EUR/MWh_FT	2015
	capture rate	0.9	per unit	
	carbondioxide-input	0.326	t_CO_2/MWh_FT	
	efficiency	0.799	per unit	2017
	electricity-input	0.007	MWh_el/MWh_FT	
	hydrogen-input	1.421	MWh_H2/MWh_FT	
	investment	703726.446	EUR/MW_FT	2017
	lifetime	20	years	2017
Methanolisation	FOM	3	%/year	2017
	capture rate	0.9	per unit	
	carbondioxide-input	0.248	t_CO_2/MWh_MeOH	
	electricity-input	0.271	MWh_e/MWh_MeOH	
	heat-output	0.1	MWh_th/MWh_MeOH	
	hydrogen-input	1.138	MWh_H2/MWh_MeOH	
	investment	703726.446	EUR/MW_MeOH	2017
	lifetime	20	years	2017

Continued on next page

Supplementary Table 4: Summary of the main technology assumptions. (Continued)

Technology	Parameter	Value	Unit	Currency year
Shipping fuel methanol	CO_2 intensity	0.2482	tCO_2/MWh_th	2020
	fuel	72	EUR/MWh_th	2020
Hydrogen-store	FOM	0.43	%/year	2020
	investment	4779.9527	EUR/MWh	2020
	lifetime	30	years	2020
Hydrogen (g) pipeline	FOM	3.1667	%/year	2015
	electricity-input	0.019	MW_e/1000km/MW_H2	2015
	investment	303.6845	EUR/MW/km	2023
	lifetime	50	years	2015
Hydrogen (g) pipeline repurposed	FOM	3.1667	%/year	2015
	electricity-input	0.019	MW_e/1000km/MW_H2	2015
	investment	129.4682	EUR/MW/km	2023
	lifetime	50	years	2015
Hydrogen (l) storage tank	FOM	2	%/year	2015
	investment	793.7456	EUR/MWh_H2	2015
	lifetime	20	years	2015
Hydrogen (l) transport ship	FOM	4	%/year	2019
	capacity	11000	t_H2	2019
	investment	393737000	EUR	2019
	lifetime	20	years	2019
Hydrogen evaporation	FOM	2.5	%/year	2020
	investment	146.8405	EUR/kW_H2	2022
	lifetime	20	years	2015
Hydrogen liquefaction	FOM	2.5	%/year	2020
	electricity-input	0.203	MWh_el/MWh_H2	
	hydrogen-input	1.017	MWh_H2/MWh_H2	
	investment	889.9426	EUR/kW_H2	2022
	lifetime	20	years	2022
Hydrogen pipeline	FOM	3	%/year	2015
	investment	282.5452	EUR/MW/km	2015
	lifetime	40	years	2015
HVAC overhead	FOM	2	%/year	2011
	investment	442.1414	EUR/MW/km	2011
	lifetime	40	years	2011
HVDC overhead	FOM	2	%/year	2011
	investment	442.1414	EUR/MW/km	2011
	lifetime	40	years	2011
HVDC submarine	FOM	0.35	%/year	2018
	investment	1008.2934	EUR/MW/km	2017
	lifetime	40	years	2018
Electricity distribution grid	FOM	2	%/year	2015
	investment	529.1108	EUR/kW	2015
	lifetime	40	years	2015
	FOM	2	%/year	2015
	investment	148.151	EUR/kW	2015
	lifetime	40	years	2015

Supplementary References

[1] Neumann, F., Zeyen, E., Victoria, M. & Brown, T. The potential role of a hydrogen network in Europe. *Joule* **7**, 1793–1817 (2023).

[2] Rahdan, P., Zeyen, E., Gallego-Castillo, C. & Victoria, M. Distributed photovoltaics provides key benefits for a highly renewable European energy system. *Applied Energy* **360**, 122721 (2024).

[3] *Documentation of PyPSA: Python for Power System Analysis* (PYPSA Developers). https://pypsa.readthedocs.io/en/latest/.

[4] *PYPSA, Energy System Technology Data* (PYPSA Developers). https://github.com/PyPSA/technology-data.

[5] Zeyen, E., Victoria, M. & Brown, T. Endogenous learning for green hydrogen in a sector-coupled energy model for europe. *Nature communications* **14**, 3743 (2023).

[6] National emissions reported to the UNFCCC and to the EU Greenhouse Gas Monitoring Mechanism, April 2024. European Environment Agency (EEA) (2024). https://doi.org/10.2909/6331f651-8863-4656-a911-669f2a332a1e. (2024).

[7] Provisional UK greenhouse gas emissions national statistics 2023. Tech. Rep., Department for Energy Security and Net Zero https://www.gov.uk/government/statistics/provisional-uk-greenhouse-gas-emissions-national-statistics-2023. (2024).

[8] Victoria, M., Zhu, K., Brown, T., Andresen, G. B. & Greiner, M. Early decarbonisation of the European energy system pays off. *Nature communications* **11**, 1–9 (2020).

[9] DEA: Technology Data for Generation of Electricity and District Heating. Danish Energy Agency (2024). https://ens.dk/en/analyses-and-statistics/technology-data-generation-electricity-and-district-heating. (2024).

[10] *kWh Face Off! East-West Vs South* Autarco Sénat https://www.autarco.com/en/updates/kwh-face-off-east-west-vs-south. 2022).

[11] Ong, S., Campbell, C., Denholm, P., Margolis, R. & Heath, G. Land-Use Requirements for Solar Power Plants in the United States. National Renewable Energy Laboratory (NREL) https://www.osti.gov/biblio/1086349. (2013).

[12] Huld, T., Müller, R. & Gambardella, A. A new solar radiation database for estimating PV performance in Europe and Africa. *Solar energy* **86**, 1803–1815 (2012).

[13] Pfeifroth, U., Kothe, S. & Trentmann, J. Validation Report Meteosat Solar Surface Radiation and Effective Cloud Albedo Climate Data Record SARAH-2. Tech. Rep., EUMETSAT Satellite Application Facility on Climate Monitoring https://www.cmsaf.eu/SharedDocs/Literatur/document/2016/saf_cm_dwd_val_meteosat_hel_2_1_pdf.html. (2016).

[14] Hersbach, H. *et al.* The ERA5 global reanalysis. *Quarterly Journal of the Royal Meteorological Society* **146**, 1999–2049 (2020).

[15] Szabo, L. *et al.* Impacts of large-scale deployment of vertical bifacial photovoltaics on European electricity market dynamics. *Nature Communications* **15**, 6681 (2024).

[16] Photovoltaic Geographical Information System (PVGIS). https://joint-research-centre.ec.europa.eu/photovoltaic-geographical-information-system-pvgis/getting-started-pvgis/pvgis-user-manual_en.

[17] Alkan, S. & Ates, Y. Pilot Scheme Conceptual Analysis of Rooftop East–West-Oriented Solar Energy System with Optimizer. *Energies* **16**, 2396 (2023).

[18] TYNDP: Ten-Year Network Development Plan. Tech. Rep., ENTSO-E https://tyndp-data.netlify.app/tyndp2018/. (2018).

[19] Gawlick, J. & Hamacher, T. Impact of coupling the electricity and hydrogen sector in a zero-emission European energy system in 2050. *Energy Policy* **180**, 113646 (2023).

[20] Gøtske, E. K., Andresen, G. B., Neumann, F. & Victoria, M. Designing a sector-coupled European energy system robust to 60 years of historical weather data. *Nature Communications* **15**, 1–12 (2024).

[21] *Documentation of atlite: A Lightweight Python Package for Calculating Renewable Power Potentials and Time Series* (atlite Developers). https://atlite.readthedocs.io/en/latest/.

[22] Akar, S. *et al.* 2020 Annual Technology Baseline (ATB) Cost and Performance Data for Electricity Generation Technologies. National Renewable Energy Laboratory (NREL) https://doi.org/10.7799/1644189. (2020).

[23] Bogdanov, D. *et al.* Radical transformation pathway towards sustainable electricity via evolutionary steps. *Nature communications* **10**, 1–16 (2019).

[24] Breyer, C. *et al.* Reflecting the energy transition from a European perspective and in the global context—Relevance of solar photovoltaics benchmarking two ambitious scenarios. *Progress in Photovoltaics: Research and Applications* **31**, 1369–1395 (2023).

[25] IRENA Renewable Power Generation Costs in 2021 . Tech. Rep., International Renewable Energy Agency, Abu Dhabi https://www.irena.org/-/media/Files/IRENA/Agency/Publication/2022/Jul/IRENA_Power_Generation_Costs_2021.pdf. (2022).

[26] IRENA Renewable Power Generation Costs in 2022 . Tech. Rep., International Renewable Energy Agency, Abu Dhabi https://www.irena.org/-/media/Files/IRENA/Agency/Publication/2023/Aug/IRENA_Renewable_power_generation_costs_in_2022.pdf. (2023).

[27] Cherp, A., Vinichenko, V., Tosun, J., Gordon, J. A. & Jewell, J. National growth dynamics of wind and solar power compared to the growth required for global climate targets. *Nature Energy* **6**, 742–754 (2021).
